# Spirolones A–E, five spiroketals from a productive saline soil derived *Penicillium raistrickii*

**DOI:** 10.3389/fmicb.2024.1495396

**Published:** 2024-11-22

**Authors:** Desheng Liu, Liying Ma, Xianguo Rong, Huihui Kang, Weizhong Liu

**Affiliations:** Laboratory of Natural Drug Discovery and Research, College of Pharmacy, Binzhou Medical University, Yantai, China

**Keywords:** azaphilone-based spiroketal, saline soil-derived fungus, *Penicillium raistrickii*, antibacterial effect, cytotoxicity

## Abstract

**Background:**

In recent decades, spiroketals which features with two rings joined by a spiro atom, have stimulated a great deal of researches for their diverse significant biological activities. Fungi are proved to be key reservoirs of structurally unique chemical skeletons. Up to date, [6,6]-spiroketals from fungal strains are still sporadically reported. Guided by UV information based on HPLC-DAD system, five unreported azaphilone-based [6,6]-spiroketals **(1-5)**, named spirolones A-E, along with one known analogue, pestafolide A **(6)**, were isolated from a productive saline soil derived fungus *Penicillium raistrickii*.

**Methods:**

Their planar structures were solved through a comprehensive set of spectroscopic techniques including UV, IR, HRESIMS and 1D/2D NMR. The absolute configurations were determined by X-ray single-crystal diffractions or the modified Mosher’s method. The misassignment of absolute configuration of pestafolide A was revised by X-ray crystallographic analysis in this study. All the isolated compounds were screened for antibacterial effect against Pseudomonas aeruginosa, Staphylococcus aureus and Escherichia coli based on the microplate approach and the cytotoxicity towards HepG2 and Hep3B cell lines based on MTT assay.

**Results and discussion:**

The results suggested that compound **4** displayed some activity against P. aeruginosa and E. coli with MIC values of 35.00 μg/mL and 55.00 μg/mL, with MIC values of the positive control of streptomycin at 12.50 μg/mL and 8.00 μg/mL, respectively. Compound **5** exhibited inhibitory effect against HepG2 cell line with IC50 value of 3.40 μM, and 8.14 μM against Hep3B cell line with doxorubincin as positive control. Compounds **4** and **5** may be capable of contribution for the development of new antibiotics or antitumor agents.

## Introduction

Spiroketals have been being a hotspot of natural product chemistry since its debut in 1930s, when the first generation of spiroketals, steroidal saponins and sapogenins, made its debut from the plants in Mexico or the United States (Perron and Albizati, 1989). Up to now, hundreds of spiroketals with diverse structural scaffolds and biological properties have been reported (Zhang et al., 2018). Recently, a new research tide on natural occurring spiroketals has been surged up especially since 2015, due to the inspiration that the Nobel Prize was rewarded to William C. Cambell for the discovery of avermectin, a spiroketal motif bearing compound (Campbell, 2012).

[5,5]-spiroketal, [5,6]-spiroketal and [6,6]-spiroketal ring systems constitute the vast majority of the general spiroketal chemistry. Of them, [6,6]-spiroketals, existed as terpenes, polyketides, macrolides, or the miscellaneous forms of the above, are very impressive for their amazing extensive activities, especially in the pharmaceutical fields, such as anti-HIV, antibacterial, antifungal, anti-CHIKV, anti-parasitic, cytotoxic, antioxidant activities, as well as vasorelaxant effect, inhibitors of TTCC (T-type voltage-gated calcium channels), Na^+^/K^+^–ATPase and isocitrate lyase, etc. (Zhang et al., 2018; Verano and Tan, 2017; Sperry et al., 2010; Gillard and Brimble, 2019). Now, spiroketals has attracted intensive attention from chemical and pharmacological communities. Consequently, continuous investigation about the isolation, chemical characterization, pharmacological evaluation with great enthusiasm are being carried out on them.

In our previous search of structurally unique and biologically active natural products from saline soil derived fungi, five new azaphilone-based spiroketals, including benzannulate [6,6]-spiroketals and benzo-fused 2,8-dioxabicyclo[3.3.1]nonane moiety bearing [6,6]-spiroketals, have been purified and identified from a talent fungus (Ma et al., 2012; Liu et al., 2014). Preliminary bioactivity screening result confirmed them with cytotoxic activity, and inhibitory effect of the growth of developing oocytes in the sea urchin *Strongylocentrotus intermedius*. Further investigations revealed that peneciraistin C might be a potential lead compound due to its ability to trigger apoptotic and autophagic lung cell death (Liu et al., 2014; Pan et al., 2013).

As part of our ongoing interest in spiroketal chemistry through cytotoxic activity guided screening and valuable information from fingerprint figures based on HPLC-DAD system, the saline soil derived *Penicillium raistrickii* displayed UV absorptions of the chromatographic peaks which were resemble to those of spiroketal skeletons in our early published papers. Herein, the details of isolation, structural elucidation and the cytotoxicity effect of the isolated spiroketals are reported.

## Materials and methods

### General experimental procedures

An uncorrected XRC-1 micro-melting apparatus was applied to measure the melting points. An Autopol Plus digital polarimeter was used to determine the optical rotations. A TU-1091 spectrophotometer and a Chirascan spectropolarimeter were used to record UV and ECD spectra, respectively. A Nicolet 6700 spectrophotometer by an attenuated total reflectance (ATR) approach was introduced to obtain IR spectra. A Bruker Avance 400 with TMS as the internal standard was used to record NMR data at 400 MHz and 100 MHz for ^1^H and ^13^C, respectively. A Bruker Smart 1,000 CCD X-ray diffractometer and a 1200RRLC-6520 Accurate-Mass Q-TOF LC/MS mass spectrometer was applied to determine crystal structure and HRESIMS data in this work, respectively. A SHIMADZU LC-6 AD Liquid Chromatography with an SPD-20A Detector on an ODS column [HyperClone 5 μm ODS (C_18_) 120 Å, 250 × 10 mm, Phenomenex, 4 mL/min] was employed for the semipreparative HPLC purification.

Silica gel (200–300 mesh, Qingdao Marine Chemical Inc., China), reversed-phase C_18_ silica gel (Pharmacia Fine Chemical Co., Ltd., Uppsala, Sweden) and Sephadex LH-20 (Ge Healthcare Bio-Sciences AB, Uppsala, Sweden) were used for column chromatography (CC). All solvents used for CC were of analytical grade (Tianjin Fuchen Chemical Reagents Co., Ltd.), and the solvents used for HPLC were of HPLC grade (Merck & Co Inc).

### Fungal materials, culture conditions, fermentation, extraction and purification

*P. raistrickii* was isolated from saline soil samples collected from Circum-Bohai-Sea region in Zhanhua county with a GenBank Accession NO. HQ717799, and the fungal strain was kept in laboratory of natural drug discovery and research, Binzhou Medical University.

The fermentation and extraction protocol were performed as described in the previous paper (Ma et al., 2019). The whole culture broth afforded 15.8 g crude extract, which was subsequently subjected to a silica gel liquid column eluting with different solvents in a gradient of increasing polarity from petroleum ether, ethyl acetate to MeOH to yield six fractions (Fr.s 1–6) based on TLC analyses. Fr.2 (1.2 g) was further chromatographed on a LH20 column eluting with aqueous MeOH to afford five subfractions (Fr.s 2.1–2.5). Fr.s 2.3 (113 mg) was loaded on a normal silica gel column eluting with chloroform/MeOH (20:1) as solvent system, followed by preparative TLC chromatography to yield compound **1** (10.2 mg). Fr.3 (1.8 g) was further separated on a normal phase silica gel column with a step gradient of petroleum ether/ethyl acetate (3:1 to 1:1, v/v) to give six fractions (Fr.s 3.1–Fr.s 3.6). Fr.s 3.4 was reloaded on a Sephadex LH-20 column to remove the pigments with 100% MeOH as mobile phase. Then, purification was carried out on a semipreparative HPLC with MeOH/0.2% trifluoroacetic acid (TFA) aqueous solution (v/v) (6:4, 4 mL/min) to yield compound **2** (14.2 mg, t*_R_* = 11.5 min) and **5** (20.4 mg, t*_R_* = 16.4 min). Fr.4 (2.2 g) was fractionated on a Sephadex LH-20 column with 100% MeOH as the eluent to afford seven subfractions (Fr.s 4.1–4.7). Among them, Fr.s 4.3 (137.3 mg) was subjected to a normal silica gel column with chloroform/methanol (15:1) as mobile phase, followed by purification on a semipreparative HPLC system with MeOH/0.2%TFA aqueous solution (v/v) (6:4, 4 mL/min) as mobile phase to yield compound **3** (10.8 mg, t*_R_* = 8.4 min) and **6** (21.3 mg, t*_R_* = 14.6 min), respectively. Similarly, Fr. 4.4 was passed through a normal silica gel column with chloroform/methanol (15:1) as eluent. Purification was subsequently carried out on a semipreparative HPLC with MeOH/0.2% TFA aqueous solution (v/v) (6:4, 4 mL/min) as mobile phase to yield **4** (12.7 mg, t*_R_* = 11.2 min).

Spirolone A (**1**): colorless needles (MeOH); mp 165–167°C; ${\left[\alpha \right]}_{D}^{20}$ −110.48 (*c* 0.062, MeOH); IR (ATR) *ν*_max_: 3,142, 2,966, 2,937, 1,624, 1,514, 1,470, 1,435, 1,339, 1,240, 1,105, 1,033, 994, 983, 918, 792, 741, 711, 678 cm^−1^. UV (MeOH) *λ*_max_ (log *ε*): 276 (4.01), 219 (4.17) nm. HRESIMS *m*/*z* 295.1173 [M+H]^+^ (calc for C_15_H_19_O_6_, 295.1176).

Spirolone B (**2**): colorless blocks; mp 164–166°C; ${\left[\alpha \right]}_{D}^{20}$ −36.3 (*c* 0.040, MeOH); IR (ATR) *ν*_max_: 3,230, 2,919, 1,622, 1,512, 1,435, 1,320, 1,239, 1,161, 1,105, 1,089, 1,055, 989, 971, 916, 843, 753, 673 cm^−1^. UV (MeOH) *λ*_max_ (log *ε*): 275 (4.02), 219 (4.22) nm. ECD (MeOH) *λ*_max_ (Δ*ε*): 315 (0.8358), 276 (−3.8092), 237 (−0.9145). HRESIMS *m*/*z* 293.1024 [M+H]^+^ (calc for C_15_H_17_O_6_, 293.1020).

Spirolone C (**3**): colorless needles; mp 144–146°C; ${\left[\alpha \right]}_{D}^{20}$ −92.89 (*c* 0.10, MeOH); IR (ATR) *ν*_max_: 3,465, 2,943, 2,869, 1,647, 1,460, 1,241, 1,407, 1,384, 1,297, 1,192, 1,141, 1,058, 989, 969, 926, 880, 818, 776, 736 cm^−1^. UV (MeOH) *λ*_max_ (log *ε*): 244 (3.92) nm. ECD (MeOH) *λ*_max_ (Δ*ε*): 350 (−1.9070), 307 (+0.8084), 267 (−2.8336), 209 (+3.1835). HRESIMS *m*/*z* 305.1358 [M+Na]^+^ (calc for C_15_H_22_O_5_Na, 305.1359).

Spirolone D (**4**): colorless needles; mp 156–158°C; ${\left[\alpha \right]}_{D}^{20}$ −182.06 (*c* 0.10, MeOH); IR (ATR) *ν*_max_: 3,351, 2,933, 1,649, 1,403, 1,294, 1,192, 1,158, 1,142, 1,057, 990, 938, 913, 851, 830, 753, 669 cm^−1^. UV (MeOH) *λ*_max_ (log *ε*): 242 (3.86) nm. ECD (MeOH) *λ*_max_ (Δ*ε*): 350 (−1.9467), 307 (+2.6462), 269 (−3.8314), 209 (+0.6098). HRESIMS *m*/*z* 321.1303 [M+Na]^+^ (calc for C_15_H_22_O_6_Na, 321.1309).

Spirolone D (**5**): colorless amorphous powder; ${\left[\alpha \right]}_{D}^{20}$ −184.34 (*c* 0.10, MeOH); IR (ATR) *ν*_max_: 1,742, 1,678, 1,631, 1,443, 1,406, 1,371, 1,217, 1,067, 1,027, 998, 935, 916, 889, 852, 826, 678 cm^−1^. UV (MeOH) *λ*_max_ (log *ε*): 236 (3.88) nm. ECD (MeOH) *λ*_max_ (Δ*ε*): 327 (−2.7045), 263 (−12.8365), 211 (+1.3999). HRESIMS *m*/*z* 363.1415 [M+Na]^+^ (calc for C_17_H_24_O_7_Na, 363.1414).

Pestafolide A (**6**): ${\left[\alpha \right]}_{D}^{20}$ −87.32 (*c* 0.11, MeOH); ECD (MeOH) λ_max_ (Δ*ε*): 350 (− 2.5878), 306 (+0.9354), 266 (−6.2737), 208 (+2.6118).

### X-ray crystal structure analysis of 1, 2, 3 and 6

The X-ray crystallographic data were recorded on a Bruker Smart 1,000 CCD X-ray diffractometer with graphite monochromated Cu Kα carried out in the *φ*-*ω* scan mode. The structure was solved by direct methods with the SHELXTL software package, and expanded using difference Fourier techniques, refined by the program SHLXL-97 and the full-matrix least-squares calculations. The crystallographic data for the structure of **1**, **2**, **3** and **6** have been deposited with the Cambridge Crystallographic Data Centre as supplementary publication CCDC 940798. These data can be obtained free of charge from the Cambridge Crystallographic Data Centre via www.ccdc.cam.ac.uk/data_request/cif.

Crystal data for spirolone A (**1**): 2C_15_H_18_O_6_·CH_3_OH; *Mr* = 620.63, Orthorhombic, space group P2(1)2(1)2(1), *a* = 11.5508(7) Å, *b* = 14.7480(7) Å, *c* = 18.5255(11) Å, *α* = *β* = *γ* = 90°, *V* = 3155.8(3) Å^3^, *Z* = 4, *T* = 293(2) K, *μ* (Cu Kα) = 0.857 mm^−1^, *D_calc_* = 1.306 g/cm^3^, *F* (000) = 1,320, 20,062 reflections measured (7.64 ≤ 2Θ ≤ 132.36), 5,528 unique (*R_int_* = 0.0665) which were used in all calculations. The final *R_1_* was 0.0548 [I ≥ 2σ (I)] and *wR_2_* was 0.1431 (all data). There are two identical C_15_H_18_O_6_ molecules and one methanol molecule in the asymmetric unit. Flack parameter = −0.7(3).

Crystal data for spirolone B (**2**): C_15_H_18_O_6_·CH_3_OH; *Mr* = 326.34, monoclinic, space group C2, *a* = 22.6599(12) Å, *b* = 8.3767(6) Å, *c* = 9.4980(6) Å, *α* = *γ* = 90°, *β* = 114.530(2)°, *V* = 1640.14(18) Å^3^, *Z* = 4, *T* = 293(2) K, *μ* (Cu Kα) = 0.873 mm^−1^, *D_calc_* = 1.322 g/cm^3^, *F* (000) = 696, 4,918 reflections measured (8.58 ≤ 2Θ ≤ 132.02), 2,749 unique (*R_int_* = 0.0160) which were used in all calculations. The final *R_1_* was 0.0357 [I ≥ 2σ (I)] and *wR_2_* was 0.1007 (all data). Flack parameter = 0.08(16).

Crystal data for spirolone C (**3**): C_15_H_22_O_5_·H_2_O; *Mr* = 300.34, Orthorhombic, space group P2(1)2(1)2(1), *a* = 7.1173(10) Å, *b* = 9.9391(9) Å, *c* = 22.0570(18) Å, *α* = *β* = *γ* = 90°, *V* = 1560.3(3) Å^3^, *Z* = 4, *T* = 293(2) K, *μ* (Cu Kα) = 0.817 mm^−1^, *D_calc_* = 1.279 g/cm^3^, *F* (000) = 648, 8,998 reflections measured (8.02 ≤ 2Θ ≤ 132.06), 2,727 unique (*R_int_* = 0.1257) which were used in all calculations. The final *R*_1_ was 0.0882 [I ≥ 2σ (I)] and *wR*_2_ was 0.2250 (all data). Flack parameter = 0.1(8).

Crystal data for pestafolide A (**6**): C_15_H_22_O_5_·H_2_O; *Mr* = 300.34, Monoclinic, space group P2(1), *a* = 14.3449(4) Å, *b* = 8.0701(2) Å, *c* = 14.5721(4) Å, *α* = *γ* = 90°, *β* = 111.414(3)°, *V* = 1570.48(7) Å^3^, *Z* = 4, *T* = 293(2) K, *μ* (Cu Kα) = 0.812 mm^−1^, *D_calc_* = 1.270 g/cm^3^, *F* (000) = 648, 5,229 reflections measured (6.52 ≤ 2Θ ≤ 132.10), 2,959 unique (*R_int_* = 0.0199) which were used in all calculations. The final *R_1_* was 0.0361 [I ≥ 2σ (I)] and *wR_2_* was 0.0957 (all data). Flack parameter = 0.3(2).

### Preparation of MTPA esters of spirolone D (4)

Compounds **4a** and **4b** (MTPA esters of **4**) were prepared through the approach as reported previously (Ma et al., 2012). Compound **4** (2 mg) was dissolved in pyridine (0.5 mL), followed by adding (*R*)-MTPA chloride (20 μL) and DMAP (50 μg). The mixture was shaken throughly and carefully. Then the reaction was terminated by addition of 1 mL water after stir for 12 h at 28°C. The mixture was extracted with 2 mL ethyl acetate three times. The ethyl acetate phase was further evaporated under reduced pressure. The residue was purified on HPLC system with an ODS column (MeOH/H_2_O, 90:10, 4 mL/min) to obtain the (*S*)-MTPA ester (**4a**). In the similar fashion, the (*S*)-MTPA ester (**4b**) was achieved with (*R*)-MTPA chloride.

Data for compoud **4a**: ^1^H NMR (CDCl_3_, 400 MHz) *δ*_H_ 5.36 (1H, dd, *J* = 10.1, 6.0 Hz, H-5), 4.87 (1H, t, *J* = 2.6, H-10), 4.49 (1H, d, *J* = 17.04 Hz, H-9a), 4.04 (1H, d, *J* = 14.32 Hz, H-9b), 3.80 (1H, m, H-13), 2.46 (1H, dd, *J* = 18.7, 5.6 Hz, H-4a), 2.35 (1H, m, H-4b), 2.22 (1H, m, 11a), 1.89 (1H, m, H-2a), 1.84 (1H, m, H-2b), 1.74 (1H, m, H-11b), 1.52 (1H, m, H-12a), 1.35 (1H, m, H-12b), 1.25 (3H, s, H-15), 1.08 (3H, d, *J* = 6.2, H-14).

Data for compoud **4b**: ^1^H NMR (CDCl_3_, 400 MHz) *δ*_H_ 5.38 (1H, dd, *J* = 10.2, 6.0 Hz, H-5), 4.87 (1H, t, *J* = 2.6, H-10), 4.51 (1H, d, *J* = 16.12 Hz, H-9a), 4.05 (1H, d, *J* = 16.84 Hz, H-9b), 3.77 (1H, m, H-13), 2.52 (1H, dd, *J* = 17.6, 5.9 Hz, H-4a), 2.34 (1H, m, H-4b), 2.17 (1H, m, 11a), 2.03 (1H, m, H-2a), 1.94 (1H, m, H-2b), 1.87 (1H, m, H-11b), 1.37 (1H, m, H-12a), 1.21 (1H, m, H-12b), 1.26 (3H, s, H-15), 1.04 (3H, d, *J* = 6.2, H-14).

### Biological assay

*Pseudomonas aeruginosa*, *Staphylococcus aureus*, and *Escherichia coli* were introduced to evaluate the antibacterial activity of the isolated compounds on the base of the microplate approach. DMSO and streptomycin were applied as the negative control and the positive control, respectively. The antibacterial biological assay was completed according to the method as previously reported (Ma et al., 2019).

The cytotoxicity toward HepG2 cell line based on MTT assay of the isolated compounds were performed in conformity with methods as previously described in our group (Gao et al., 2017; Xie et al., 2019).

## Results and discussion

**Figure 1:**
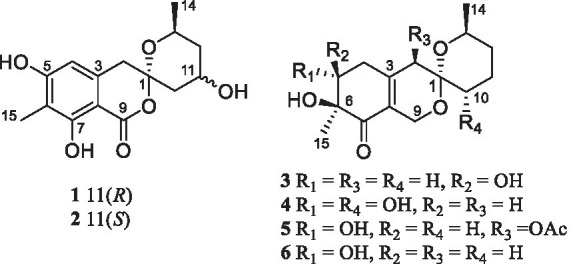
Structure of compounds **1**–**6**.

Chemical investigation of *P. raistrickii* led to the isolation of five new azaphilone-based spiroketal compounds (**1–5**) and one known compound (**6**) (Figure 1).

Spirolone A (**1**) was isolated as colorless needles. The molecular formula, C_15_H_18_O_6_, with 7 degrees of unsaturation, was determined by a molecular ion peak at *m*/*z* 293.1021 [M+H]^+^ (calcd for C_15_H_17_O_6_, 293.1020) in its positive HRESIMS spectrum (Supplementary Figure S1). Its UV spectrum exhibited an absorption maximum at 276 and 221 nm (Supplementary Figure S3). The IR spectrum displayed absorption bands of hydroxyl groups (3,141 cm^−1^) and conjugated ester carbonyl (1,624 cm^−1^), respectively. The ^1^H NMR spectrum (Table 1, Supplementary Figure S1) of **1** showed a hydroxyl proton at *δ*_H_ 11.37, an aromatic proton at *δ*_H_ 6.29, two oxymethines at *δ*_H_ 3.98 and 3.87, three diastereotopic methylenes at *δ*_H_ 3.20 (H-2a)/2.94 (H-2b), 2.25 (H-10a)/1.40 (H-10b), and 1.95 (H-12a)/1.10 (H-12b), and two methyl groups at *δ*_H_ 1.95 and 1.02. The ^13^C NMR spectrum (Table 1) showed 15 well-resolved resonances, which were further classified by DEPT and HSQC experiments as one ester carbonyl, six aromatic (one methine and five quaternaries), one ester carbonyl, two oxymethine, three methylene, along with two methyl carbons. All the described data accounted for its 1D NMR resonances and required it to be tricyclic.

**Table 1:** ^1^H and ^13^C NMR data for **1**–**3** (recorded at 400 MHz for ^1^H and 100 MHz for ^13^C).

No	1^a^		2^a^		3^b^	
	*δ*_C_	*δ*_H_ (*J* in Hz)	*δ*_C_	*δ*_H_ (*J* in Hz)	*δ*_C_	*δ*_H_ (*J* in Hz)
1	104.9, C		103.9, C		94.9, C	
2	37.5, CH2	3.20, d (16.6) 2.94, d (16.6)	38.7^*^	3.12, d (16.5) 2.87, d (16.5)	41, CH_2_	2.37, d (18.9) 2.12, d (18.9)
3	136.6, C		136.5, C		148.5, C	
4	106.7, CH	6.29, s	106.5	6.27, s	34.8, CH_2_	2.66, br d (19.3) 2.52, br d (19.2)
5	162.6, C		162.1, C		72.8, CH	4.13 dd, (1.1, 1.9)
6	108.5, C		108.4, C		75.8, C	
7	161, C		160.9, C		198.5, C	
8	98.8, C		99.2, C		126.0, C	
9	168.4, C		168.8, C		56.8, CH_2_	4.53, br d (15.5) 4.05, br d (15.5)
10	42.5, CH_2_	2.25, dd (13.1, 3.8) 1.40, dd (13.1, 11.7)	38.34^*^, CH_2_	2.08, brd (15.0) 1.75, dd (15.0, 3.9)	33.7, CH_2_	1.70, br d (13.2) 1.50, dt, (13.4, 4.4)
11	61.8, CH	3.98, m	62.4	4.03, m	18.9, CH_2_	1.92, m; 1.61, m
12	41.7, CH_2_	1.95, m 1.10, q (11.8)	38.29^*^, CH_2_	1.65, brd (13.6) 1.40, td (13.6, 2.7)	32.2, CH_2_	1.61, m; 1.18, m
13	67.2, CH	3.87, m	61.4, CH	4.30, m	66.9, CH	3.78, m
14	21.2, CH_3_	1.02, d (6.2)	21.1, CH_3_	1.00, d (6.3)	21.7, CH_3_	1.09, d (6.3)
15	7.7, CH_3_	1.95, s	7.6, CH_3_	1.95, s	23.1, CH_3_	1.34, s
OH-4				10.48, s		
OH-7		11.37, s		11.48, s		
OH-11				4.64, d (2.5)		

^a^Recorded in DMSO; ^b^Recorded in CDCl_3_; ^*^Exchangeable signal.

**Figure 2:**
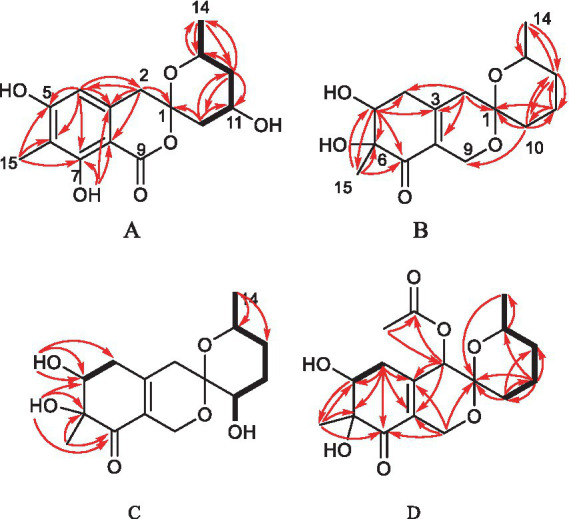
Selected HMBC and COSY correlations of **1** and **2** (A), **3** (B), **4** (C) and **5** (D).

One aromatic methine and five aromatic quaternary carbons suggested the existence of a pentasubstituted phenyl ring in **1**. The positions of the substituents around the phenyl ring was deduced from the following data. The upfield chemical shift of the methyl group at aromatic ring (*δ*_C_ 7.7) suggested that the corresponding aromatic carbon which it was anchored on located between two oxygenated aromatic ones, which was further confirmed by the HMBC correlations from H-15 to C-5, C-6 and C-7 (Figure 2 and Supplementary Figure S9). The downfield chemical shift of the hydroxyl hydrogen (*δ*_H_ 11.37) implied that the chemical shift was influenced by the nearby ester carbonyl, which led to the conclusion that they were *ortho*-positioned. Furthermore, the placement of the downfield methylene (C-2, *δ*_C_ 43.6) on the phenyl ring was determined by the HMBC correlations from H-2 to C-3, C-4 and C-8. The ^1^H–^1^H COSY spectrum disclosed the spin system from H-10 to H-14, constructing the partial structure from C-12 to C-14 as shown in Figure 2. Finally, the HMBC correlations from H-2 and H-10 to C-1, the remaining oxygen element, and the remaining unsaturation completed the gross structure of **1** to be a miscellaneous [6,6]-spiroketal.

**Figure 3:**
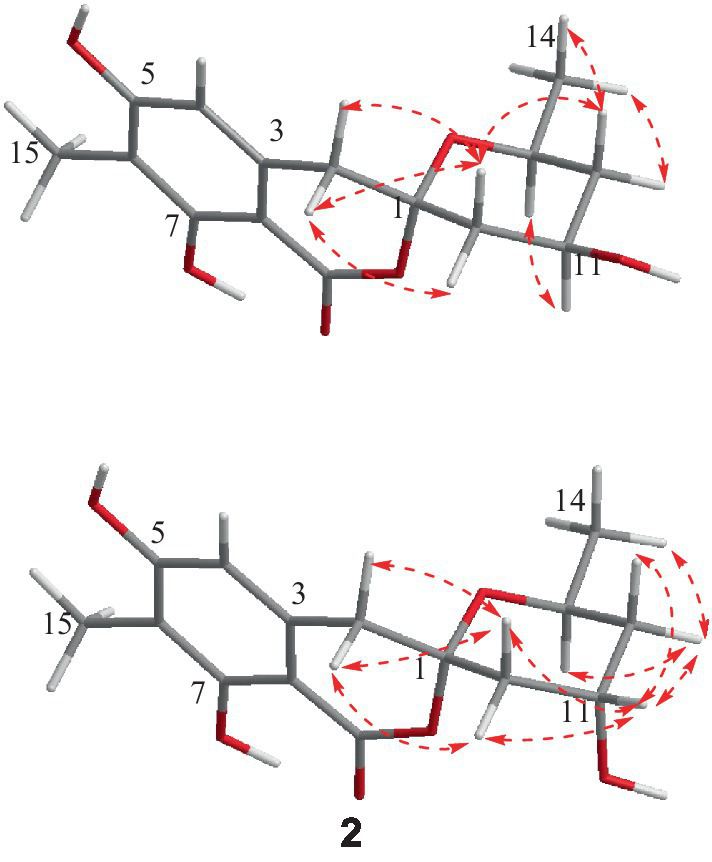
Key NOESY correlations for compounds **1** and **2**.

**Figure 4:**
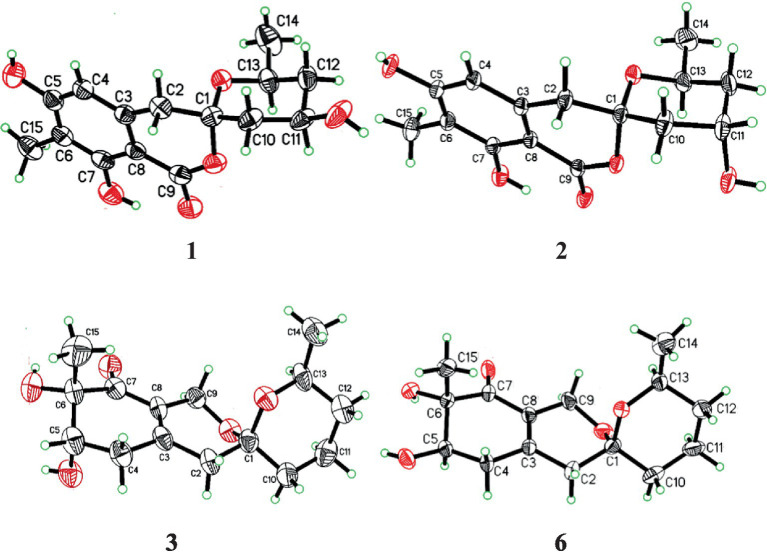
X-ray ORTEP drawing of compound **1**, **2**, **3** and **6**.

The relative configuration of **1** was deduced according to NOESY (Supplementary Figure S10) correlations and resonanable *J*-based analysis. The 1,3-diaxial NOESY correlation between H-11 and H-13, together with the NOESY correlations of H-14 with H-12a and 12b (Figure 3), implied that both the methyl and the hydroxyl on the tetrahydropyran ring were in an equatorial orientation. The NOESY correlations between H-10b and H-12b, along with the large coupling constants of *J*_H-11, H-10b_ (11.7 Hz) and *J*_H-11, H-12b_ (11.8 Hz), indicated that the protons of H-10b and H-12b were in an axial position opposite to H-11. The NOESY correlations of H-2a with H-10a and H-10b, and of H-2b with H-10b, proved that the methylene (C-2) to be equatorial. All the diagnostic NOESY data determined the stereochemistry at C-1 and C-13 to be both of *S* absolute configuration (Isaka et al., 2014, p. 11; Farimani and Miran, 2014). Finally, the absolute configuration of **1** was further proved by single crystal X-ray diffraction analyses to be 1*S*, 11*R*, 13*S* (Figure 4).

Spirolone B (**2**) was also obtained as colorless needles, and it shared the same molecular formula with **1** (C_15_H_18_O_6_, 7 degrees of unsaturation) as indicated by the negative HRESIMS spectrum (Supplementary Figure S11). The almost identical IR and UV spectra of **1** and **2** demonstrated their structural similarities. The 1D- and 2D-NMR data (Supplementary Figures S15-S19) proved it had the same plane structure with that of **1**. Detailed comparison of the corresponding chemical shifts between **1** and **2** revealed significant differences at C-10, C-12, and C-13, which were adjacent to C-11 asymmetric center, demonstrating an opposite configuration at C-11 with that of **1**. Furthermore, the NOESY correlations of H-14 with H-12a and H-12b, and H-12a with H-13, suggested H-13 and H-12a were on the trans axial positions (Figure 3 and Supplementary Figure S20), which was supported by the td multiplicity (*J* = 14.3, 2.9 Hz) of H-12b. Further NOESY correlations of H-11 with H-10a, H-10b, H-12a and H-12b proved the hydroxyl was on axial orientation. The relative configuration of the spiroketal carbon was determined by the same NOESY correlations as those in **1**. Finally, the single crystal X-ray diffraction analyses confirmed the absolute configuration of **2** to be 1*S*, 11*S*, 13*S* (Figure 4).

Spirolone C (**3**), a colorless needle, was assigned the molecular formula C_15_H_22_O_5_, with 5 degrees of unsaturation on the basis of the positive-ion HRESIMS peak at *m/z* 305.1358 [M+Na]^+^ (Supplementary Figure S21) calcd for C_15_H_22_O_5_Na, 305.1359). The IR spectrum absorption bands indicated the presence of hydroxyl groups (3,465 cm^−1^) and one α, β-unsaturated ketone carbonyl group (1,946 cm^−1^), respectively. The UV spectrum exhibited maximum absorption peak at 244 nm. Analysis of the NMR spectroscopic data (Supplementary Figures S25-S28) disclosed the presence of two methyl groups (*δ*_H/C_ 1.09, 3H, d, *J* = 6.3 and 1.34/21.7; 3H, s/23.1, respectively), six methylene groups (*δ*_H/C_ 1.92, 1H, m and 1.61, 1H, m/18.9; 1.61, 1H, m and 1.1, 1H, m/32.2; 1.70, 1H, br d, *J* = 13.2 Hz and 1.50, 1H, dt, *J* = 13.4, 4.4 Hz, respectively; 2.66, 1H, br d, *J* = 19.3 Hz and 2.52, 1H, br d, *J* = 19.3 Hz/34.8; 2.37, 1H, d, *J* = 18.9 Hz and 2.12, 1H, d, *J* = 18.9 Hz/41.0; 4.53, 1H, br d, *J* = 15.5 Hz and 4.05, 1H, br d, *J* = 15.5 Hz/56.8), two oxymethines (*δ*_H/C_ 3.78, 1H, m/66.9; 4.13, 1H, dd, *J* = 1.1 and 1.9/72.8, respectively), five quaternary carbons including one oxygenated carbon (*δ*_C_ 75.8), one spiroketal carbon (*δ*_C_ 94.9), two olefenic carbons (*δ*_C_ 126.0 and 148.5) and one carbonyl carbon (*δ*_C_ 198.5).

**Figure 5:**
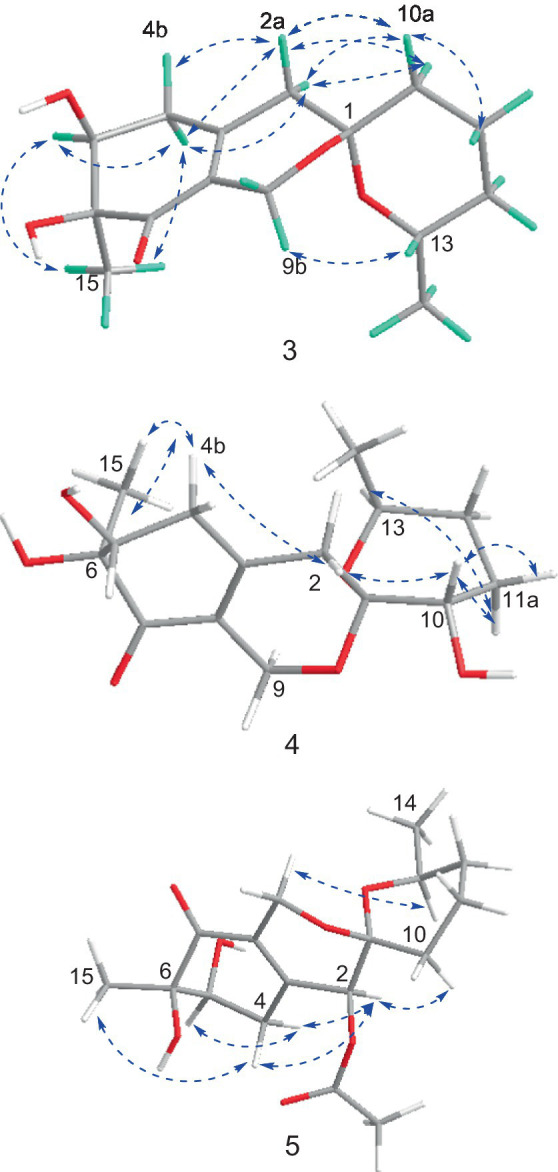
Key NOSEY correlations of compounds **3**, **4** and **5**.

The NMR data of **3** were highly similar to those of **6**, which was also isolated in the present study, indicating that they occupied the same gross structure. The relative configuration of **3** was assigned by the rational analysis of the NOESY data (Figure 5 and Supplementary Figure S29). The NOESY correlations between H_3_-15 and H-5, H-5 and H-4a suggested that H-4a, H-5 and H_3_-15 were on the same face with respect to the corresponding ring system, whereas correlations between H-9b and H-13 illustrated the relative configuration of C-1 and C-13. Moreover, detailed comparison of the chemical shifts disclosed obvious differences existed in the C and H atoms around the C-5 chiral center (Table 2), which implied that C-5 possessed an opposite configuration to that of compound **6**. The absolute configuration of C-6 was assigned by the ECD spectrum (Supplementary Figure S24) comparing with that of **6**, which unambiguously defined an *S* configuration of it, and subsequently assigned an *S* configuration of C-5, based on the information mentioned above. The absolute configuration of C-1 and C-13 was confirmed by the single X-ray diffraction (Flack parameter, 0.0(8)) (Figure 4). Therefore, the absolute configuration of **3** was established as 1*S*, 5*S*, 6*S* and 13*S*.

**Table 2:** ^1^H and ^13^C NMR data for **4**–**6** (^1^H, 400 MHz; ^13^C, 100 MHz; *δ* in ppm, TMS).

No.	4^a^		5^b^		6^a^	
	*δ*_C_	*δ*_H_ (*J* in Hz)	*δ*_C_	*δ*_H_ (*J* in Hz)	*δ*_C_	*δ*_H_ (*J* in Hz)
1	96.4, C		96.2, C		95, C	
2	37.5, CH_2_	2.34, d (19.7) 2.19, d (19.7)	68.4, CH	5.14 (s)	41, CH_2_	2.33, d (19.1) 2.21, d (19.1)
3	149.7, C		145.6, C		150.9, C	
4	36.6, CH_2_	2.50, m 2.17, m	33.2, CH_2_	2.61, dd (18.5, 5.1) 2.33, m	36.1, CH_2_	2.53, m 2.41, m
5	71.4, CH	3.75^c^	71.8, CH	4.00, dd (10.3, 5.7)	72, CH	4.02^c^
6	75.9, C		77.7, C		77.6, C	
7	198.9, C		200.1, C		199.2, C	
8	125.9, C		130.4, C		127.1, C	
9	56.6, CH_2_	4.21, d (15.2) 3.92, d (15.2)	57.5, CH_2_	4.51, bd (16.6) 4.04, bd (16.6)	57, CH_2_	4.45, d (15.5) 4.02^c^
10	66.4, CH	3.31, m	29, CH_2_	1.73, bd (13.2) 1.35, dt, (13.2, 4.5)	33.7, CH_2_	1.69, m 1.52, m
11	26.6, CH_2_	1.93, m 1.56, m	18.3, CH_2_	1.83, m 1.65, m	18.8, CH_2_	1.92, m 1.59^c^
12	25.8, CH_2_	1.51, m 1.26, m	31.9, CH_2_	1.61, m 1.20, m	32.1, CH_2_	1.59^c^ 1.19, m
13	65.9, CH	3.68, m	68.4, CH	3.71, m	66.9, CH	3.77, m
14	21.6, CH_3_	1.02, d (6.2)	21.4, CH_3_	1.09, d (6.2)	21.6, CH_3_	1.10, d (6.0)
15	18.1, CH_3_	1.08, s	17.3, CH_3_	1.29, s	17.7, CH_3_	1.28 (s)
16			170.4, C			
17			20.6, CH_3_	2.16, s		
OH-5		5.10, s	170.4, C			4.02^c^
OH-6		5.10, s	20.6, CH_3_	2.16, s		4.02^c^
OH-10		4.87, d (5.4)				

^aRecorded in DMSO; bRecorded in CDCl3; cOverlapping signals.^

Spirolone D (**4**) was isolated as colorless powder. Its molecular formula was assigned to be C_15_H_22_O_6_ on the basis of the NMR data and the positive HRESIMS data, which exhibited a pseudomolecular ion peak at *m*/*z* 321.1309 [M+Na]^+^ (Supplementary Figure S30, calcd for C_15_H_22_O_6_Na, 321.1303) with 16 *amu* more than that of **3**. And the IR and UV spectrum of **4** (Supplementary Figures S31, S32) exhibited similar absorption bands with that of **3**, indicating that it was an analogue with one more oxygen atom. The ^1^H- and ^13^C-NMR (Supplementarys Figures S34, S35) data revealed the highly structural similarity to **3**. The obvious difference was the presence of one hydroxyl group (*δ*_H_ 4.87, 1H, d, *J* = 5.4) and an oxymethine (*δ*_H/C_ 3.31, 1H, m/66.4) signals, and the absence of a methylene signal compared with **3**. The location of the hydroxyl was determined by the HMBC (Supplementary Figure S37) correlations from the hydroxyl proton to C-1, 10 and 11, respectively.

The relative configuration of **4** were solved by NOESY experiments (Figure 5 and Supplementary Figure S40). NOESY spectrum exhibited interactions between protons of H_3_-15 and H-4b, H-4b and H-2a, H-2a and H-10, indicating that these protons were spatially on the same side. The same conclusion could also be reached when it came to protons of H-9b and H-13, H-13 and H-11a. Likewise, it could be supposed that H_3_-15 and H-5 were on the opposite side of the ring system since there were no NOESY interactions between them. In addition, it happened again that NOESY correlations occurred between H-9b and H-13 as it did in compound **3**, which determined the corresponding relative configuration of C-1 and C-13 and dropped a hint of the absolute configuration to be the same as compound **3**.

The absolute configuration of C-6 was confirmed by ECD (Supplementary Figure S33) excitation chirality. The ECD spectrum of 4 exhibited an obvious positive Cotton effect at 218 nm and an obvious positive effect at 249 nm which was almost identical to compounds **3** and **6**, indication the S absolute configuration at C-6, and then assigned an *R* configuration of C-5, based on the rational analysis of NOESY interaction information given above.

**Figure 6:**
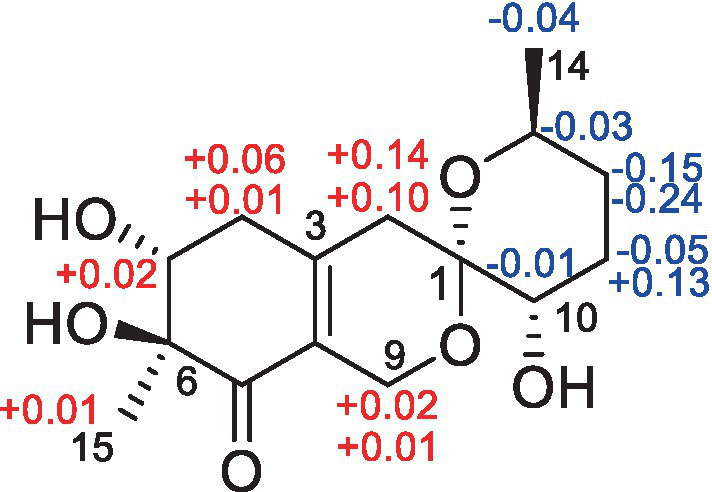
Δ*δ* values (in ppm) = *δ_S_* − *δ_R_* obtained for (*R*)- and (*S*)-MTPA esters **4a** and **4b**.

The modified Mosher’s method ultimately determined the absolute configuration at C-10 in **4**. Mosher’s ester analysis confirmed that (−)-enantiomer of 4 to be the 10*R* configuration and (+)-enantiomer of 4 to be the 10*S* configuration. The differences of chemical shifts Δδ_*S*-*R*_ in ^1^H NMR were calculated and determined the chiral center of C-10 to be *S* configurations (Figure 6). Finally, all the information combined with the NOESY interactions between H-9b and H-13 confirmed the absolute configuration of **4** was established as 1*S*, 5*R*, 6*S*, 10*S* and 13*S*.

Spirolone E (**5**) was also obtained as colorless amorphous powder. Its molecular formula was assigned to be C_17_H_24_O_7_ on the basis of the NMR data as well as the positive HRESIMS data, which exhibited a pseudomolecular ion peak at *m*/*z* 363.1435 [M+Na]^+^ (Supplementary Figure S43) calcd for C_17_H_24_O_7_Na, 363.1413) with 58 *amu* more than that of **3**. The IR and UV absorption behavior of **5** (Supplementary Figures S44, S45) highly resembled with those of **3**, which suggested their structure similarity. Detailed comparison of the 1D NMR data of the two compounds disclosed the presence of one methyl (*δ*_H/C_ 2.16, 3H, s/20.6), one carbonyl (*δ*_C_ 170.4) and one oxymethine (*δ*_H/C_ 5.14, 1H, s/68.4) in **5** and the disappearance of a methylene (*δ*_H/C_ 2.37, 1H, d, *J* = 18.9 and 2.12, 1H, d, *J* = 18.9/41.0) in **3**. An acetoxy group was constructed based on the HMBC correlations from the H_3_-17 to C-16, along with the high fielded chemical shift of the latter one. Aided with the HMBC information from H2 to C-16, H_3_-17 to C-2, the acetoxy group was positioned at C-2, which was further proved by the chemical shift of C-2. So, the gross structure was determined as shown and was in accordance with all of the HSQC and HMBC information (Supplementary Figures S49, S50).

**Figure 7:**
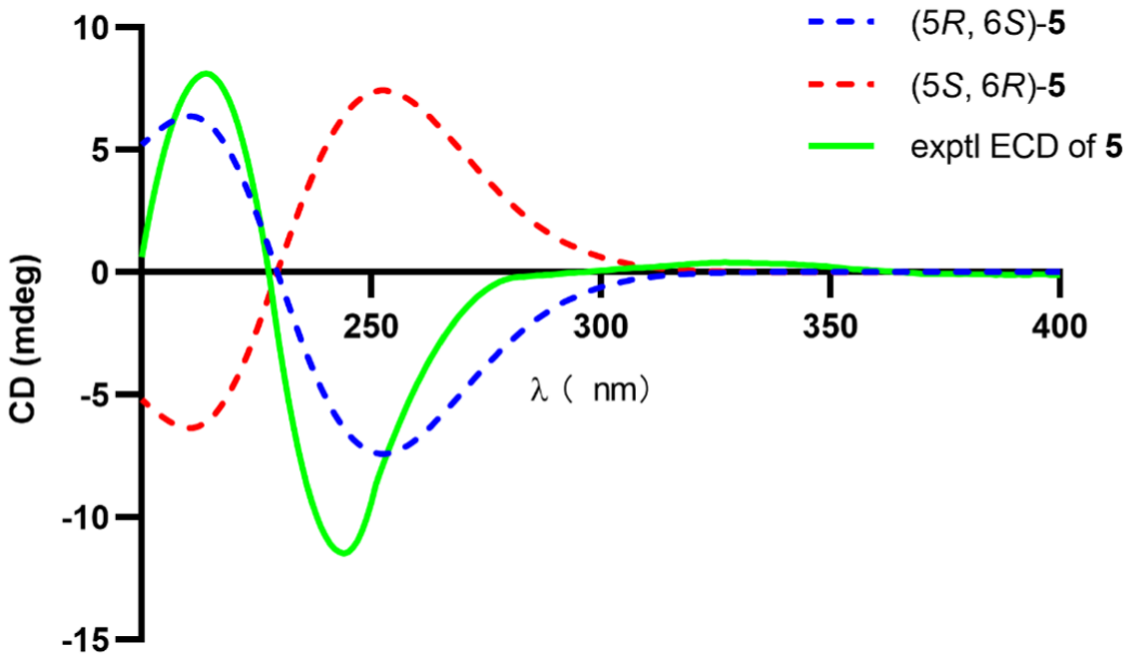
Experimental and calculated ECD of **5**.

The significant NOESY (Supplementary Figure S52) correlations of H_3_-15 with H-4b and H5 with H-4a, along without NOESY interactions between H_3_-15 and H-5 strongly suggested that they were on the opposite side of the ring. The crosspeaks between H-9b and H-13 in **5** like what compounds **3** and **4** did potently determined the relative stereochemistry of C-1 and C-13, and also provided a huge implication on the absolute configuration of it (Figure 5). Additionally, the ^1^H NMR resonance of H-10b exhibited as a triple doublet, indicating that H-10b was in an axial position. Analogously to **3** and **4**, the ECD spectrum of **5** was highly resemble to that of **6**, which ultimately established the absolute stereochemistry of C-6 as *S*, as same as what compounds **3** and **4** did. In light to the absence of NOESY interactions between H_3_-15 and H-5, the chiral center at C-5 was defined as *R* configuration. And the NOESY (Supplementary Figure S52) information between H-5 and H-4a, H-4a and H-2 provided the chiral center at C-2 as *R* configuration. Based on the relative configuration determination, ECD calculations (Figure 7) was further introduced to prove the above deduction and the stereochemistry of compound **5** was confirmed as 2*R*, 5*R*, 6*S* (Figure 4).

In addition, compound **6** was also isolated and purified in this work. The UV, IR, NMR, HRESIMS spectroscopic data, specific rotation and ECD spectrum of **6** were found to be identical to those of pestafolide A, which might have been erroneously assigned the absolute configuration of C-5. The orientations were accurately validated by single-crystal X-ray diffraction with Cu Kα radiations based on the value of the Flack parameter, 0.3(2) as 1*S*, 4*R*, 5*S* and 13*S* (Figure 4).

The bioactivities of compounds **1**–**6** were carried out in terms of antibacterial effect against of *P. aeruginosa*, *S. aureus*, and *E. coli* and cytotoxic effect against human hepatoma cells (HepG2 and Hep3B) by the microplate approach and MTT methods, respectively. According to the results (Table 3), compound **4** displayed some activity against *P. aeruginosa* and *E. coli* with MIC values of 35.00 μg/mL and 55.00 μg/mL, respectively, while the MIC values of streptomycin were 12.50 μg/mL and 8.00 μg/mL, respectively. Compound **5** exhibited a moderate cytotoxicity with an IC_50_ values of 3.40 μM against HepG2 cell line and 8.14 μM against Hep3B cell line with doxorubincin as positive control, while the others were almost inactive (IC_50_ > 10 μM).

**Table 3:** Cytotoxicity and antibacterial activities of compounds **1**–**6**.

Compounds	IC_50_ values (μM)		Minimum inhibitory concentration (MIC) (μg/mL)		
	HepG2	Hep3B	*P. aeruginosa*	*S. aureus*	*E. coli*
1	31.40 ± 0.27	45.54 ± 0.41	>100	>100	>100
2	23.54 ± 0.31	26.10 ± 0.74	>100	>100	>100
3	20.18 ± 0.045	17.34 ± 0.42	>100	>100	>100
4	35.22 ± 0.26	>50	35.00 ± 0.45	>100	55.00 ± 0.50
5	3.40 ± 0.55	8.14 ± 0.53	>100	>100	>100
6	>50	34.55 ± 0.98	>100	>100	>100
Doxorubincin	0.87 ± 0.09	1.30 ± 0.15	NT	NT	NT
Streptomycin	NT	NT	12.50 ± 0.22	8.00 ± 0.45	8.00 ± 0.30

Data are mean ± SD.

It’s a never ending pursuit for human being to seek novel bioactive small molecules to confront with endlessly emerging diseases from nature products. Marine originated fungal strains gained tremendous research focus on the chemical and biological substances. Spiro[6,6] systems are an essential subgroups of sprioketal family. They possess a unique molecular skeleton that two six-membered rings joined by a spiro atom, and the spiroketal unit plays a fundamental role in their biological value. Up to date, 6,6-sprioketals originated from the marine environment are relative rare, and most of them were produced by fungal strains (Figures 5–7).

In this study, six 6,6-sprioketals were isolated from a saline soil derived fungus. All the isolated compounds were tested for their antitumor effect against HepG2 and Hep3B cell lines, and antimicrobial activities against three pathogenic bacteria including *P. aeruginosa*, *S. aureus*, and *E. coli*. Compound **5** showed moderate to weak cytotoxic effect against two lines of human hepatoma cells, while others were weak or inactive. It seemed that acetyloxy group at C-2 position would enhance cytotoxicity of [6,6]-spiroketals. Compound **4** showed moderate activities against *P. aeruginosa* and *E. coli*. Considering several functionalities which could be modified in the framework of the isolated molecules, they were still great potential as bioactive compounds to combat pathogens or cancers.

## Conclusion

In conclusion, six spiroketals (**1**–**6**), including two benzannulated [6,6]-spiroketals and four cyclopentenone bearing [6,6]-spiroketals, were purified and identified from a talented saline soil derived fungus *P. raistrickii*, following valuable information of UV absorption based on HPLC method. Their chemical structures were solved by extensive spectroscopic approaches, X-ray diffraction analysis and ECD calculations. Interestingly, compounds **4** and **5** may be capable of contribution for the development of new antibiotics or antitumor agents.

## Data Availability

The original contributions presented in the study are publicly available. This data can be found here: [https://www.ccdc.cam.ac.uk, with deposition numbers of 2402492, 2402493, 2402494 and 2402495, respectively].
