# Supplementary material for: Spirolones A–E, five spiroketals from a productive saline soil derived Penicillium raistrickii
Source: Front Microbiol. 2024 Nov 22;15:1495396. doi: 10.3389/fmicb.2024.1495396 (PMC11621928; doi:10.3389/fmicb.2024.1495396)

checkCIF/PLATON report

Structure factors have been supplied for datablock(s) 170610a

THIS REPORT IS FOR GUIDANCE ONLY. IF USED AS PART OF A REVIEW PROCEDURE FOR PUBLICATION, IT SHOULD NOT REPLACE THE EXPERTISE OF AN EXPERIENCED CRYSTALLOGRAPHIC REFEREE.

No syntax errors found. CIF dictionary Interpreting this report

Datablock: 170610a

```
Bond precision:   C-C = 0.0094 A          Wavelength=1.54178

Cell:             a=7.1173(10)      b=9.9391(9)     c=22.0570(18)
                  alpha=90          beta=90         gamma=90
Temperature:      293 K

                Calculated                  Reported
Volume          1560.3(3)                   1560.3(3)
Space group     P 21 21 21                  P2(1)2(1)2(
Hall group      P 2ac 2ab                   ?
Moiety formula  C15 H22 O5, H2 O            ?
Sum formula     C15 H24 O6                  C15 H24 O6
Mr              300.34                      300.34
Dx,g cm-3       1.279                       1.279
Z               4                           4
Mu (mm-1)       0.817                       0.817
F000            648.0                       648.0
F000'           650.20
h,k,lmax        8,11,26                     8,11,26
Nref            2726[ 1599]                 2727
Tmin,Tmax       0.872,0.922                 0.822,0.923
Tmin'           0.815

Correction method= # Reported T Limits: Tmin=0.822 Tmax=0.923
AbsCorr = MULTI-SCAN

Data completeness= 1.71/1.00      Theta(max)= 66.030

R(reflections)= 0.0882( 1092)     wR2(reflections)= 0.2250( 2727)

S = 1.046                 Npar= 193
```

The following ALERTS were generated. Each ALERT has the format
test-name_ALERT_alert-type_alert-level.
Click on the hyperlinks for more details of the test.

Alert level C

```
RINTA01_ALERT_3_C  The value of Rint is greater than 0.12
            Rint given   0.126
STRVA01_ALERT_4_C           Flack test results are meaningless.
           From the CIF: _refine_ls_abs_structure_Flack    0.000
           From the CIF: _refine_ls_abs_structure_Flack_su    0.800
PLAT020_ALERT_3_C The value of Rint is greater than 0.12 .........      0.126 Report
PLAT026_ALERT_3_C Ratio Observed / Unique Reflections (too) Low ..         40 %
PLAT234_ALERT_4_C Large Hirshfeld Difference C4     --  C5      ..       0.18 Ang.
PLAT242_ALERT_2_C Low    'MainMol' Ueq as Compared to Neighbors of        C11 Check
PLAT340_ALERT_3_C Low Bond Precision on  C-C Bonds ...............     0.0094 Ang.
PLAT417_ALERT_2_C Short Inter D-H..H-D       H4     ..  H6C     ..       2.13 Ang.
PLAT906_ALERT_3_C Large K value in the Analysis of Variance ......      2.136 Check
PLAT978_ALERT_2_C Number C-C Bonds with Positive Residual Density.          0 Note
```

Alert level G

```
PLAT005_ALERT_5_G No Embedded Refinement Details found  in the CIF     Please Do !
PLAT007_ALERT_5_G Number of Unrefined Donor-H Atoms ..............          4 Report
PLAT032_ALERT_4_G Std. Uncertainty on Flack Parameter Value High .      0.800 Report
PLAT066_ALERT_1_G Predicted and Reported Tmin&Tmax Range Identical          ? Check
PLAT093_ALERT_1_G No s.u.'s on H-positions, Refinement Reported as      mixed Check
PLAT199_ALERT_1_G Reported _cell_measurement_temperature ..... (K)        293 Check
PLAT200_ALERT_1_G Reported   _diffrn_ambient_temperature ..... (K)        293 Check
PLAT779_ALERT_4_G Suspect or Irrelevant (Bond) Angle in CIF .... #          6 Check
              O5   -O6   -H6C     1.555   1.555   1.555             32.20 Deg.
PLAT791_ALERT_4_G The Model has Chirality at C2      (Chiral SPGR)          S Verify
PLAT791_ALERT_4_G The Model has Chirality at C6      (Chiral SPGR)          S Verify
PLAT791_ALERT_4_G The Model has Chirality at C10     (Chiral SPGR)          S Verify
PLAT791_ALERT_4_G The Model has Chirality at C11     (Chiral SPGR)          S Verify
PLAT850_ALERT_4_G Check Flack Parameter Exact Value 0.00 and s.u.        0.80 Check
PLAT899_ALERT_4_G SHELXL97   is Deprecated and Succeeded by SHELXL       2014 Note
PLAT916_ALERT_2_G Hooft y and Flack x Parameter values differ by .       0.37 Check
```

0 **ALERT level A** = Most likely a serious problem - resolve or explain
0 **ALERT level B** = A potentially serious problem, consider carefully
10 **ALERT level C** = Check. Ensure it is not caused by an omission or oversight
15 **ALERT level G** = General information/check it is not something unexpected

4 ALERT type 1 CIF construction/syntax error, inconsistent or missing data
4 ALERT type 2 Indicator that the structure model may be wrong or deficient
5 ALERT type 3 Indicator that the structure quality may be low
10 ALERT type 4 Improvement, methodology, query or suggestion
2 ALERT type 5 Informative message, check

It is advisable to attempt to resolve as many as possible of the alerts in all categories. Often the minor alerts point to easily fixed oversights, errors and omissions in your CIF or refinement strategy, so attention to these fine details can be worthwhile. In order to resolve some of the more serious problems it may be necessary to carry out additional measurements or structure refinements. However, the purpose of your study may justify the reported deviations and the more serious of these should normally be commented upon in the discussion or experimental section of a paper or in the "special_details" fields of the CIF. checkCIF was carefully designed to identify outliers and unusual parameters, but every test has its limitations and alerts that are not important in a particular case may appear. Conversely, the absence of alerts does not guarantee there are no aspects of the results needing attention. It is up to the individual to critically assess their own results and, if necessary, seek expert advice.

Publication of your CIF in IUCr journals

A basic structural check has been run on your CIF. These basic checks will be run on all CIFs submitted for publication in IUCr journals (*Acta Crystallographica*, *Journal of Applied Crystallography*, *Journal of Synchrotron Radiation*); however, if you intend to submit to *Acta Crystallographica Section C* or *E* or *IUCrData*, you should make sure that full publication checks are run on the final version of your CIF prior to submission.

Publication of your CIF in other journals

Please refer to the *Notes for Authors* of the relevant journal for any special instructions relating to CIF submission.

PLATON version of 27/03/2017; check.def file version of 24/03/2017

Datablock 170610a - ellipsoid plot

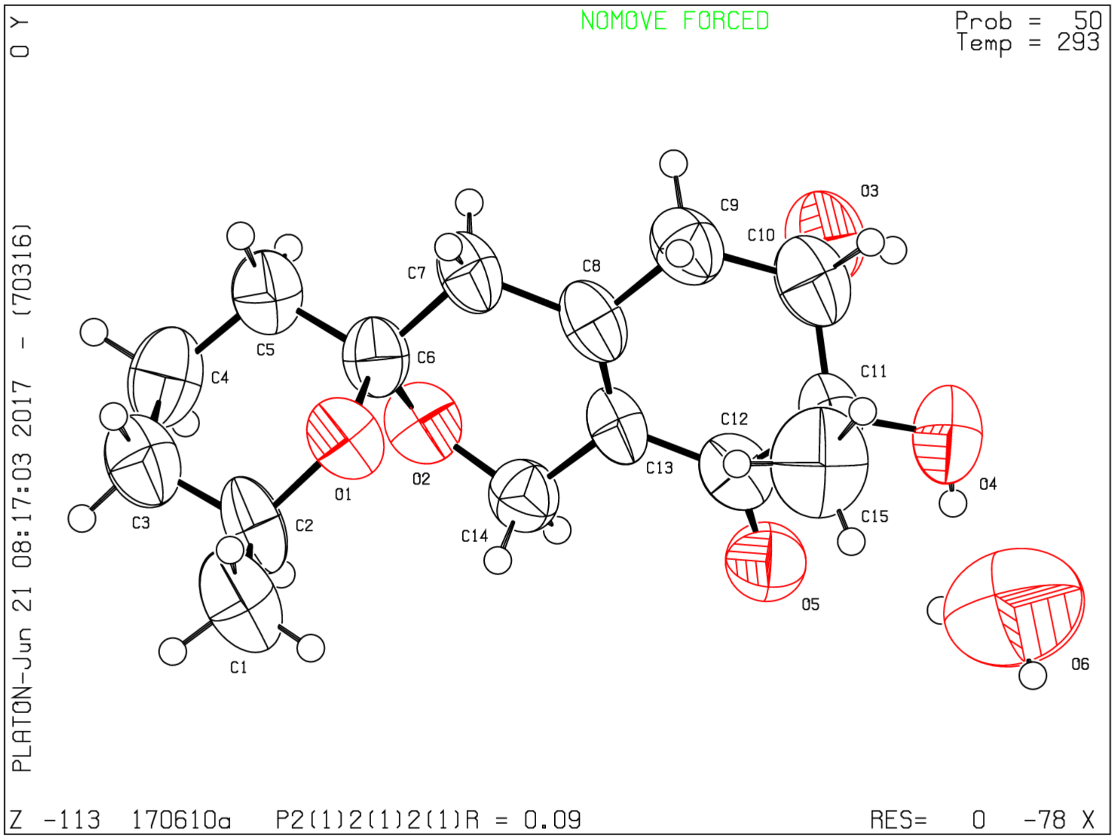

Supplement: Supplementary file 1 [file Data_Sheet_1.zip › X-ray-3/JH18-21checkcif.pdf]
