# Supplementary material for: Spirolones A–E, five spiroketals from a productive saline soil derived Penicillium raistrickii
Source: Front Microbiol. 2024 Nov 22;15:1495396. doi: 10.3389/fmicb.2024.1495396 (PMC11621928; doi:10.3389/fmicb.2024.1495396)

checkCIF/PLATON report

Structure factors have been supplied for datablock(s) 170418c

THIS REPORT IS FOR GUIDANCE ONLY. IF USED AS PART OF A REVIEW PROCEDURE FOR PUBLICATION, IT SHOULD NOT REPLACE THE EXPERTISE OF AN EXPERIENCED CRYSTALLOGRAPHIC REFEREE.

No syntax errors found. CIF dictionary Interpreting this report

Datablock: 170418c

Bond precision: C-C = 0.0043 A Wavelength=1.54178

Cell:	a=14.3449(4)	b=8.0701(2)	c=14.5721(4)
	alpha=90	beta=111.414(3)	gamma=90
Temperature:	293 K		

	Calculated	Reported
Volume	1570.48(8)	1570.48(7)
Space group	P 21	P2(1)
Hall group	P 2yb	?
Moiety formula	C15 H22 O5, H2 O	?
Sum formula	C15 H24 O6	C15 H24 O6
Mr	300.34	300.34
Dx,g cm-3	1.270	1.270
Z	4	4
Mu (mm-1)	0.812	0.812
F000	648.0	648.0
F000’	650.20	
h,k,lmax	17,9,17	17,9,17
Nref	5492[2961]	2959
Tmin,Tmax	0.753,0.735	0.732,0.748
Tmin’	0.683	

Correction method= # Reported T Limits: Tmin=0.732 Tmax=0.748
AbsCorr = MULTI-SCAN

Data completeness= 1.00/0.54 Theta(max)= 66.050

R(reflections)= 0.0361(2821) wR2(reflections)= 0.0957(2959)

S = 1.016 Npar= 384

The following ALERTS were generated. Each ALERT has the format
test-name_ALERT_alert-type_alert-level.
Click on the hyperlinks for more details of the test.

Alert level C

```
PLAT340_ALERT_3_C Low Bond Precision on  C-C Bonds ...............    0.00433 Ang.
PLAT417_ALERT_2_C Short Inter D-H..H-D       H1D     ..  H2C     ..      2.12 Ang.
PLAT911_ALERT_3_C Missing # FCF Refl Between THmin & STh/L=  0.593          2 Report
PLAT914_ALERT_3_C No Bijvoet Pairs in FCF for Non-centro Structure     Please Check
```

Alert level G

```
PLAT005_ALERT_5_G No Embedded Refinement Details found  in the CIF     Please Do !
PLAT007_ALERT_5_G Number of Unrefined Donor-H Atoms ..............          8 Report
PLAT093_ALERT_1_G No s.u.'s on H-positions, Refinement Reported as      mixed Check
PLAT199_ALERT_1_G Reported _cell_measurement_temperature ..... (K)        293 Check
PLAT200_ALERT_1_G Reported   _diffrn_ambient_temperature ..... (K)        293 Check
PLAT720_ALERT_4_G Number of Unusual/Non-Standard Labels ..........         12 Note
PLAT791_ALERT_4_G The Model has Chirality at C1A     (Chiral SPGR)          S Verify
PLAT791_ALERT_4_G The Model has Chirality at C1B     (Chiral SPGR)          S Verify
PLAT791_ALERT_4_G The Model has Chirality at C5A     (Chiral SPGR)          R Verify
PLAT791_ALERT_4_G The Model has Chirality at C5B     (Chiral SPGR)          R Verify
PLAT791_ALERT_4_G The Model has Chirality at C6A     (Chiral SPGR)          S Verify
PLAT791_ALERT_4_G The Model has Chirality at C6B     (Chiral SPGR)          S Verify
PLAT791_ALERT_4_G The Model has Chirality at C13A    (Chiral SPGR)          S Verify
PLAT791_ALERT_4_G The Model has Chirality at C13B    (Chiral SPGR)          S Verify
PLAT899_ALERT_4_G SHELXL97   is Deprecated and Succeeded by SHELXL       2014 Note
PLAT909_ALERT_3_G Percentage of Observed Data at Theta(Max) Still          90 % Note
PLAT913_ALERT_3_G Missing # of Very Strong Reflections in FCF ....          1 Note
PLAT978_ALERT_2_G Number C-C Bonds with Positive Residual Density.          6 Note
```

0 **ALERT level A** = Most likely a serious problem - resolve or explain
0 **ALERT level B** = A potentially serious problem, consider carefully
4 **ALERT level C** = Check. Ensure it is not caused by an omission or oversight
18 **ALERT level G** = General information/check it is not something unexpected

3 ALERT type 1 CIF construction/syntax error, inconsistent or missing data
2 ALERT type 2 Indicator that the structure model may be wrong or deficient
5 ALERT type 3 Indicator that the structure quality may be low
10 ALERT type 4 Improvement, methodology, query or suggestion
2 ALERT type 5 Informative message, check

It is advisable to attempt to resolve as many as possible of the alerts in all categories. Often the minor alerts point to easily fixed oversights, errors and omissions in your CIF or refinement strategy, so attention to these fine details can be worthwhile. In order to resolve some of the more serious problems it may be necessary to carry out additional measurements or structure refinements. However, the purpose of your study may justify the reported deviations and the more serious of these should normally be commented upon in the discussion or experimental section of a paper or in the "special_details" fields of the CIF. checkCIF was carefully designed to identify outliers and unusual parameters, but every test has its limitations and alerts that are not important in a particular case may appear. Conversely, the absence of alerts does not guarantee there are no aspects of the results needing attention. It is up to the individual to critically assess their own results and, if necessary, seek expert advice.

Publication of your CIF in IUCr journals

A basic structural check has been run on your CIF. These basic checks will be run on all CIFs submitted for publication in IUCr journals (*Acta Crystallographica*, *Journal of Applied Crystallography*, *Journal of Synchrotron Radiation*); however, if you intend to submit to *Acta Crystallographica Section C* or *E* or *IUCrData*, you should make sure that full publication checks are run on the final version of your CIF prior to submission.

Publication of your CIF in other journals

Please refer to the *Notes for Authors* of the relevant journal for any special instructions relating to CIF submission.

PLATON version of 27/03/2017; check.def file version of 24/03/2017

Datablock 170418c - ellipsoid plot

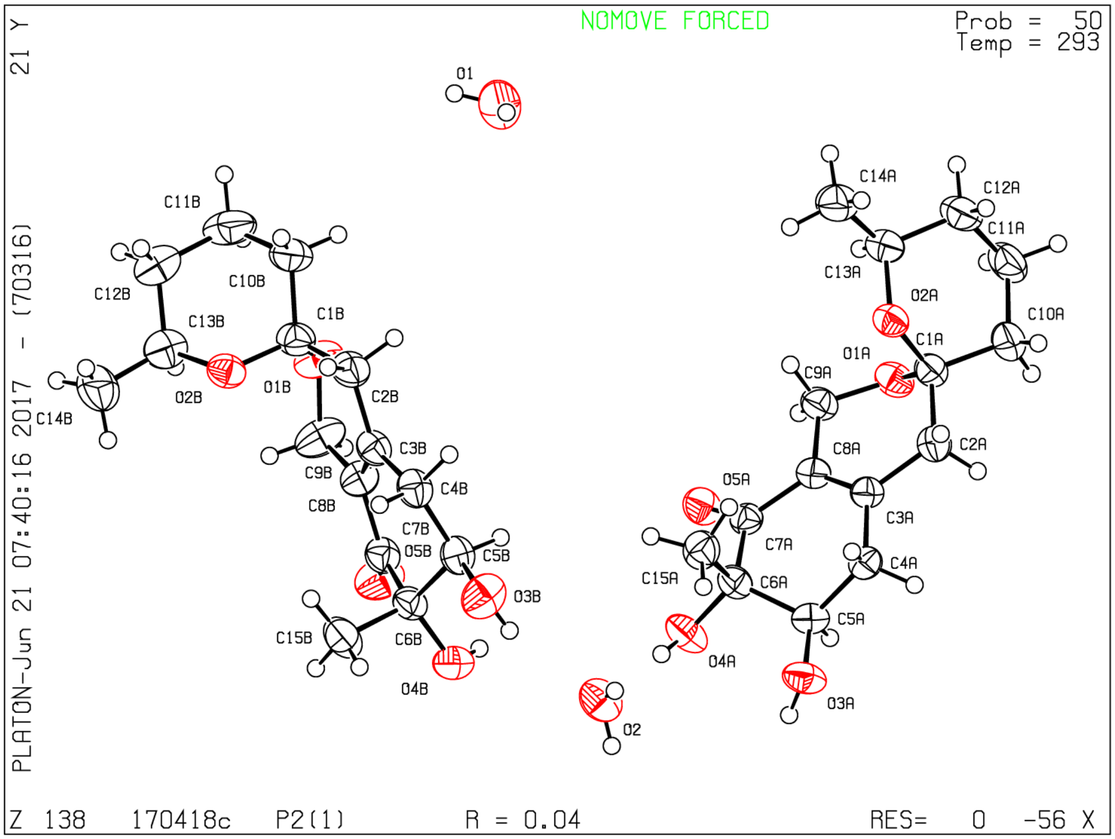

Supplement: Supplementary file 1 [file Data_Sheet_1.zip › X-ray-6/JHJ18-82checkcif.pdf]
