# Supplementary figures and images for: Spirolones A–E, five spiroketals from a productive saline soil derived Penicillium raistrickii
Source: Front Microbiol. 2024 Nov 22;15:1495396. doi: 10.3389/fmicb.2024.1495396 (PMC11621928; doi:10.3389/fmicb.2024.1495396)

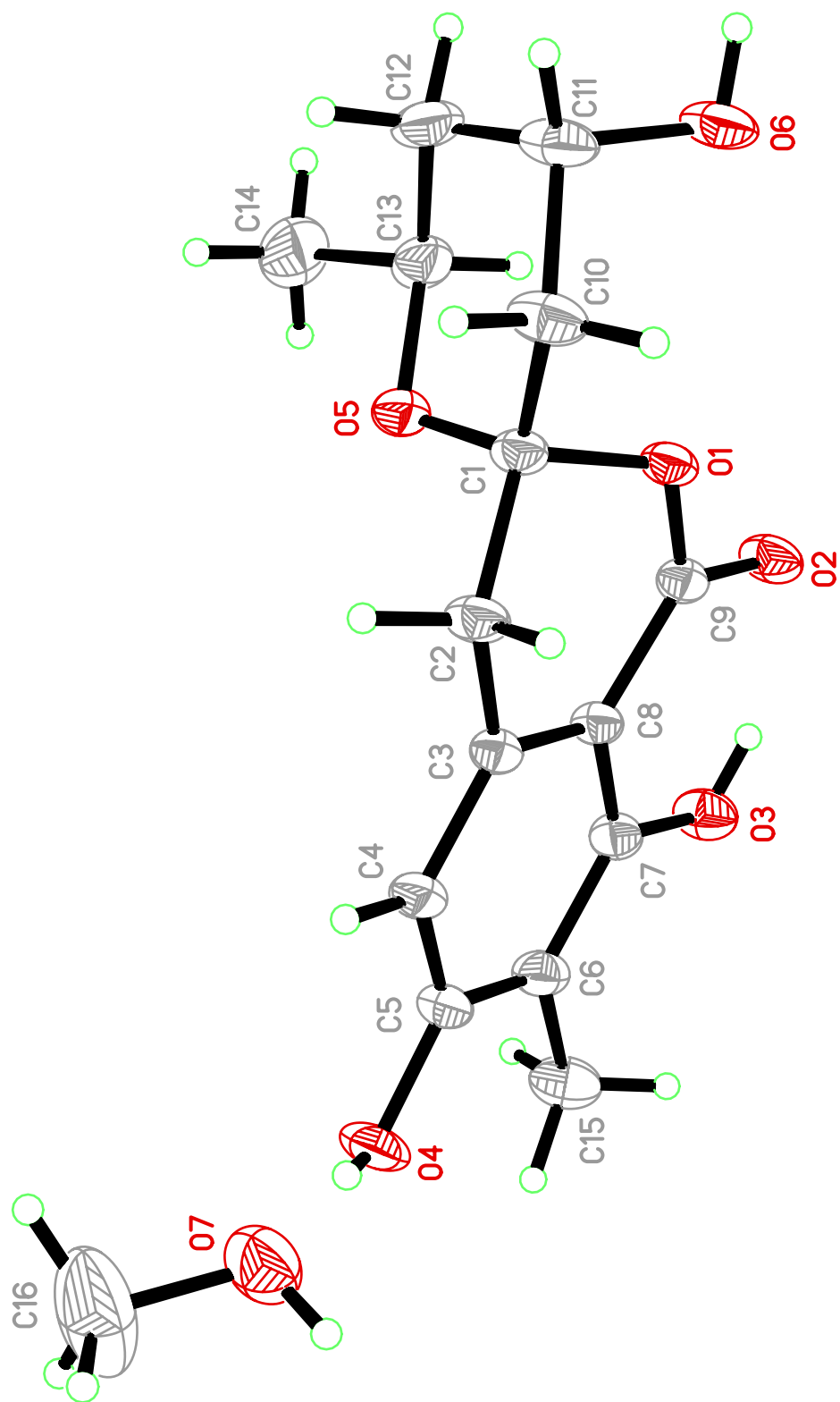

Supplement: Supplementary file 1 [file Data_Sheet_1.zip › X-ray-2/38.pdf]

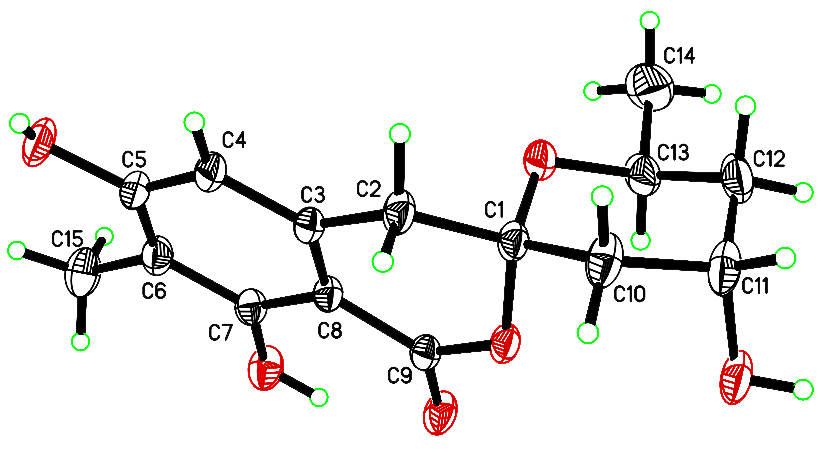

Supplement: Supplementary file 1 [file Data_Sheet_1.zip › X-ray-2/JH18-38.png]

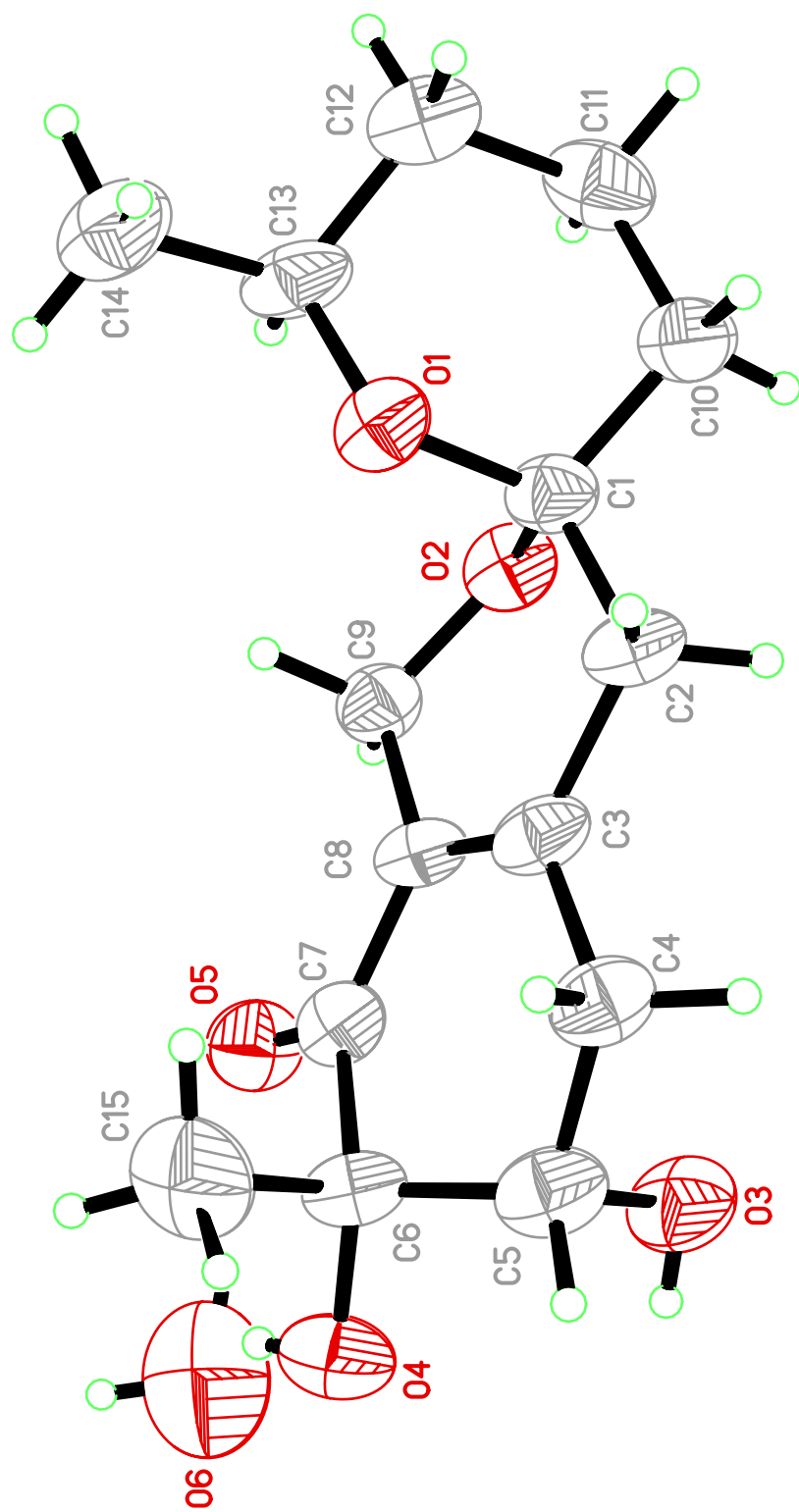

Supplement: Supplementary file 1 [file Data_Sheet_1.zip › X-ray-3/211.pdf]

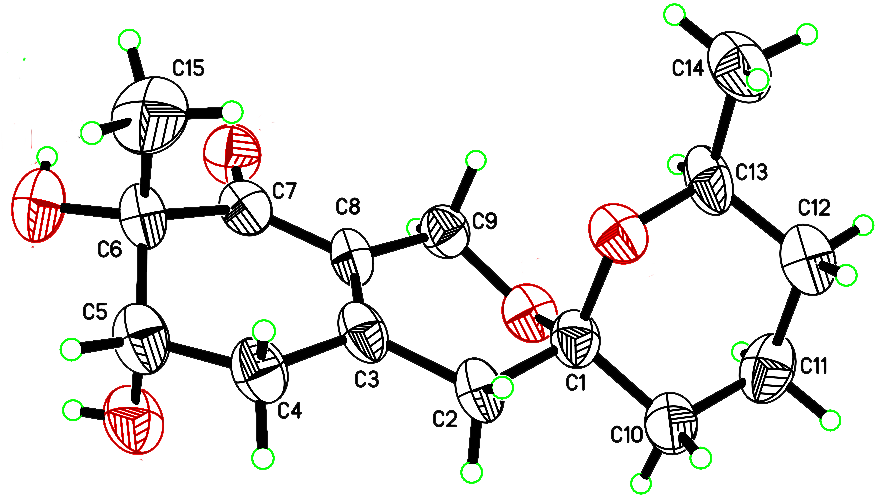

Supplement: Supplementary file 1 [file Data_Sheet_1.zip › X-ray-3/JH18-21.png]

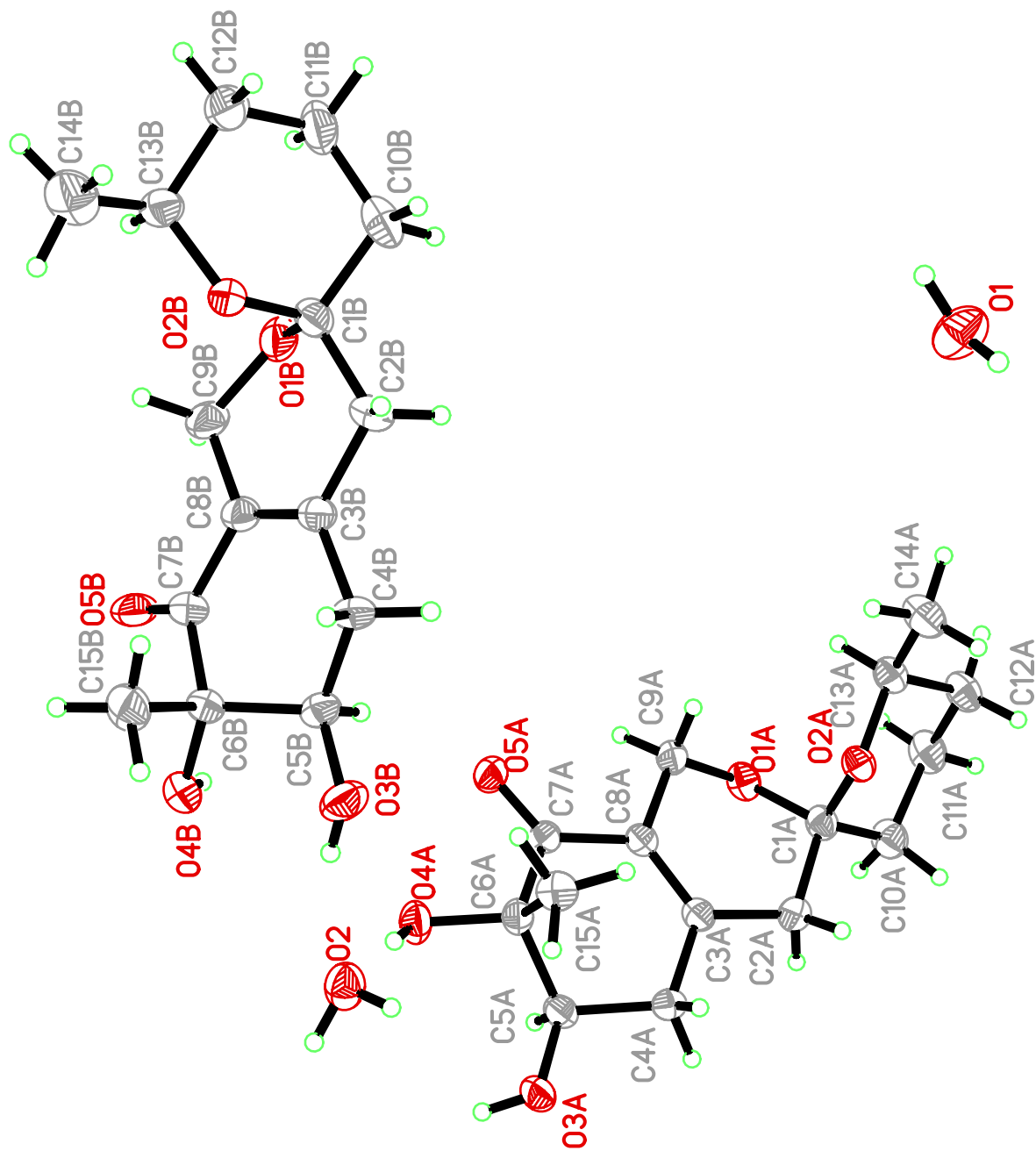

Supplement: Supplementary file 1 [file Data_Sheet_1.zip › X-ray-6/822.pdf]

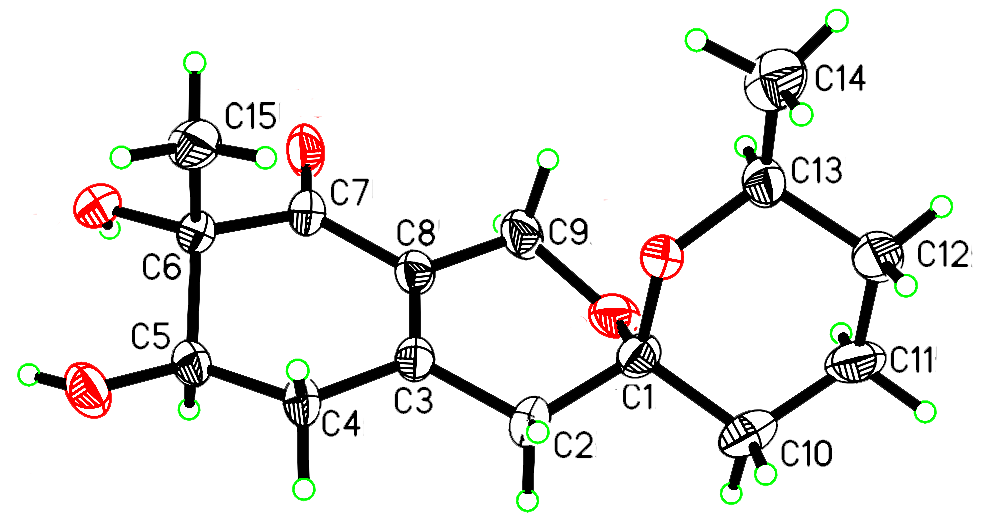

Supplement: Supplementary file 1 [file Data_Sheet_1.zip › X-ray-6/JHJ18-82.png]
