# Supplementary material for: Spirolones A–E, five spiroketals from a productive saline soil derived Penicillium raistrickii
Source: Front Microbiol. 2024 Nov 22;15:1495396. doi: 10.3389/fmicb.2024.1495396 (PMC11621928; doi:10.3389/fmicb.2024.1495396)
Supplement: Supplementary file 2 [file Data_Sheet_2.docx]

**SUPPORTING INFORMATION**

**Spirolones A-E: Five novel azaphilone-based spiroketals from a productive saline soil derived *Penicillium raistrickii***

Desheng Liu^*^, Liying Ma, Xianguo Rong, Huihui Kang, and Weizhong Liu^*^

*Laboratory of Natural Drug Discovery and Research, College of Pharmacy, Binzhou Medical University, Yantai, China*

^*^To whom correspondence should be addressed. Tel: 86-535-6913071. Fax: 86-535-6913020. E-mail: [desheng_liu@sina.com](mailto:desheng_liu@sina.com)；[lwz1963@163.com](mailto:lwz1963@163.com)

**List of supporting information**

[Figure S1. HRESIMS of spirolone A (1) 4](#_Toc179218941)

[Figure S2. IR spectrum (ATR approach) of spirolone A (1) 4](#_Toc179218942)

[Figure S3. UV spectrum (MeOH) of spirolone A (1) 5](#_Toc179218943)

[Figure S4. ECD spectrum (MeOH) of spirolone A (2) 5](#_Toc179218944)

[Figure S5. ^1^H NMR spectrum (400 MHz DMSO-*d*_6_) of spirolone A (1) 6](#_Toc179218945)

[Figure S6. ^13^C NMR spectrum (100 MHz DMSO-*d*_6_) of spirolone A (1) 6](#_Toc179218946)

[Figure S7. COSY spectrum (DMSO-*d*_6_) of spirolone A (1) 7](#_Toc179218947)

[Figure S8. HSQC spectrum (DMSO-*d*_6_) of spirolone A (1) 7](#_Toc179218948)

[Figure S9. HMBC spectrum (DMSO-*d*_6_) of spirolone A (1) 8](#_Toc179218949)

[Figure S10. NOESY spectrum (DMSO-*d*_6_) of spirolone A (1) 8](#_Toc179218950)

[Figure S11. HRESIMS of spirolone B (2) 9](#_Toc179218951)

[Figure S12. IR spectrum (ATR approach) of spirolone B (2) 9](#_Toc179218952)

[Figure S13. UV spectrum (MeOH) of spirolone B (2) 10](#_Toc179218953)

[Figure S14. ECD spectrum (MeOH) of spirolone B (2) 10](#_Toc179218954)

[Figure S15. ^1^H NMR spectrum (400 MHz DMSO-*d*_6_) of spirolone B (2) 11](#_Toc179218955)

[Figure S16. ^13^C NMR spectrum (100 MHz DMSO-*d*_6_) of spirolone B (2) 11](#_Toc179218956)

[Figure S17. COSY spectrum (DMSO-*d*_6_) of spirolone B (2) 12](#_Toc179218957)

[Figure S18. HSQC spectrum (DMSO-*d*_6_) of spirolone B (2) 12](#_Toc179218958)

[Figure S19. HMBC spectrum (DMSO-*d*_6_) of spirolone B (2) 13](#_Toc179218959)

[Figure S20. NOESY spectrum (DMSO-*d*_6_) of spirolone B (2) 13](#_Toc179218960)

[Figure S21. HRESIMS of spirolone C (3) 14](#_Toc179218961)

[Figure S22. IR of spirolone C (3) 14](#_Toc179218962)

[Figure S23. UV spectrum (MeOH) of spirolone C (3) 15](#_Toc179218963)

[Figure S24. ECD spectrum (MeOH) of spirolone C (3) 15](#_Toc179218964)

[Figure S25. ^1^H NMR spectrum (400 MHz CDCl_3_) of spirolone C (3) 16](#_Toc179218965)

[Figure S26. ^13^C NMR spectrum (100 MHz CDCl_3_) of spirolone C (3) 16](#_Toc179218966)

[Figure S27. HMBC spectrum of spirolone C (3) 17](#_Toc179218967)

[Figure S28. HSQC of spirolone C (3) 17](#_Toc179218968)

[Figure S29. NOESY spectrum of spirolone C (3) 18](#_Toc179218969)

[Figure S30. HRESIMS of spirolone D (4) 18](#_Toc179218970)

[Figure S31. IR of spirolone D (4) 19](#_Toc179218971)

[Figure S32. UV spectrum (MeOH) of spirolone D (4) 19](#_Toc179218972)

[Figure S33. ECD spectrum (MeOH) of spirolone D (4) 20](#_Toc179218973)

[Figure S34. ^1^H NMR spectrum (400 MHz DMSO-*d*_6_) of spirolone D (4) 20](#_Toc179218974)

[Figure S35. ^13^C NMR spectrum (100 MHz DMSO-*d*_6_) of spirolone D (4) 21](#_Toc179218975)

[Figure S36. DEPT spectrum of spirolone D (4) 21](#_Toc179218976)

[Figure S37. HMBC of spirolone D (4) 22](#_Toc179218977)

[Figure S38. HSQC of spirolone D (4) 22](#_Toc179218978)

[Figure S39. COSY spectrum of spirolone D (4) 23](#_Toc179218979)

[Figure S40. NOESY spectrum of spirolone D (4) 23](#_Toc179218980)

[Figure S41. ^1^H NMR spectrum (400 MHz CDCl_3_) of (*S*)-MTPA ester of 4 (4a) 24](#_Toc179218981)

[Figure S42. ^1^H NMR spectrum (400 MHz CDCl_3_) of (*R*)-MTPA ester of 4 (4b) 24](#_Toc179218982)

[Figure S43. HRESIMS of spirolone E (5) 25](#_Toc179218983)

[Figure S44. IR of spirolone E (5) 25](#_Toc179218984)

[Figure S45. UV spectrum (MeOH) of spirolone E (5) 26](#_Toc179218985)

[Figure S46. ECD spectrum (MeOH) of spirolone E (5) 26](#_Toc179218986)

[Figure S47. ^1^H NMR spectrum (400 MHz CDCl_3_) of spirolone E (5) 27](#_Toc179218987)

[Figure S48. ^13^C NMR spectrum (100 MHz CDCl_3_) of spirolone E (5) 27](#_Toc179218988)

[Figure S49. HMBC of spirolone E (5) 28](#_Toc179218989)

[Figure S50. HSQC of spirolone E (5) 28](#_Toc179218990)

[Figure S51. COSY spectrum of spirolone E (5) 29](#_Toc179218991)

[Figure S52. NOESY spectrum of spirolone E (5) 29](#_Toc179218992)

[Computation Section. 30](#_Toc179218993)


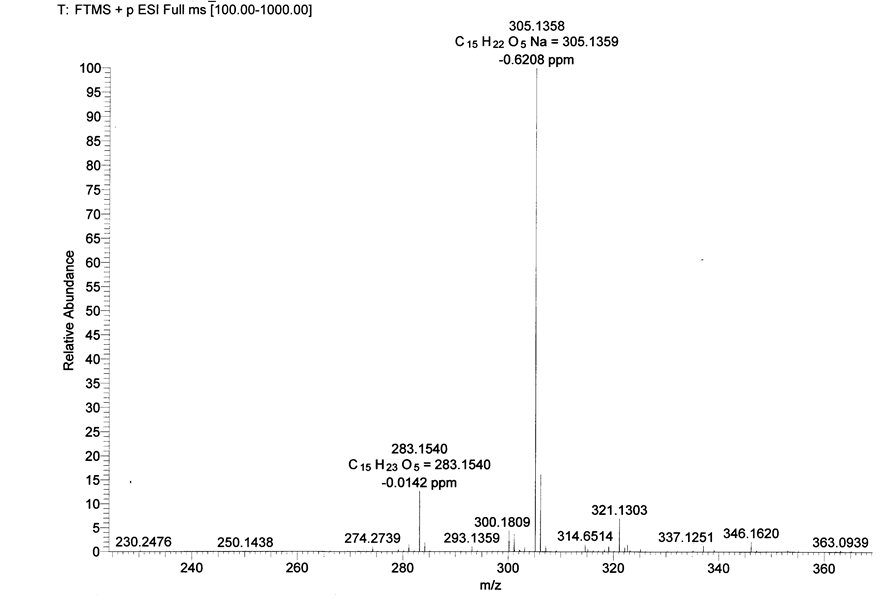


## Figure S1. HRESIMS of spirolone A (1)


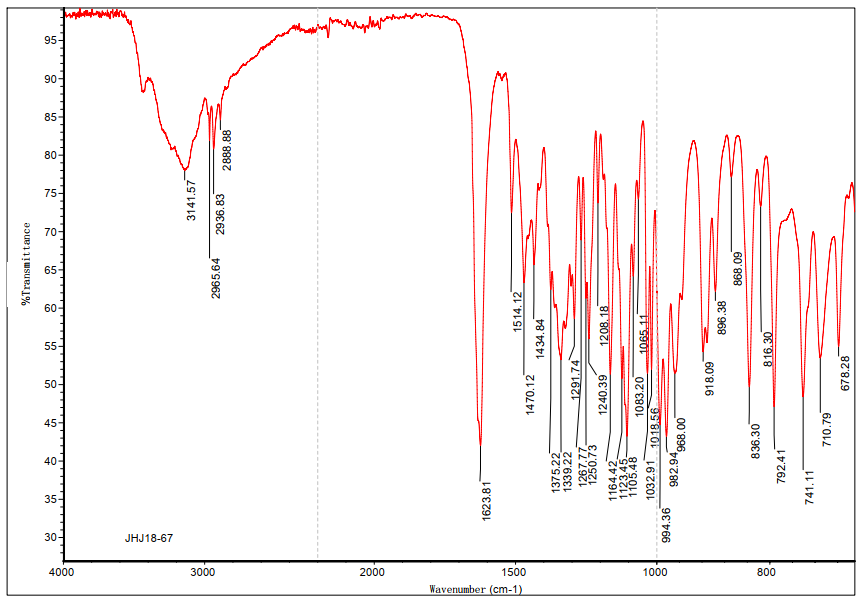


## Figure S2. IR spectrum (ATR approach) of spirolone A (1)


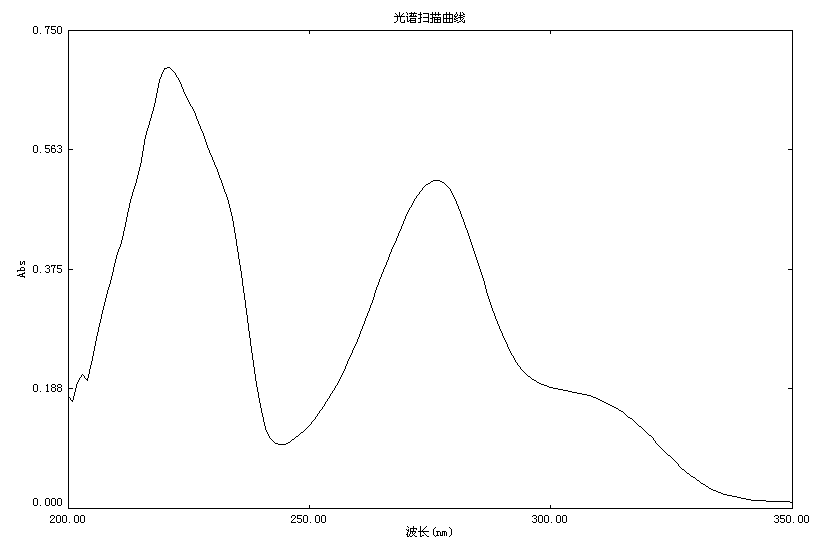


## Figure S3. UV spectrum (MeOH) of spirolone A (1)


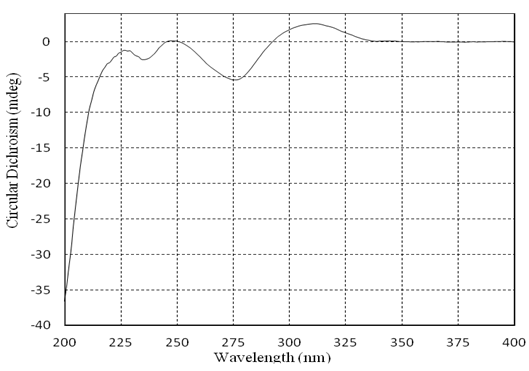


## Figure S4. ECD spectrum (MeOH) of spirolone A (2)


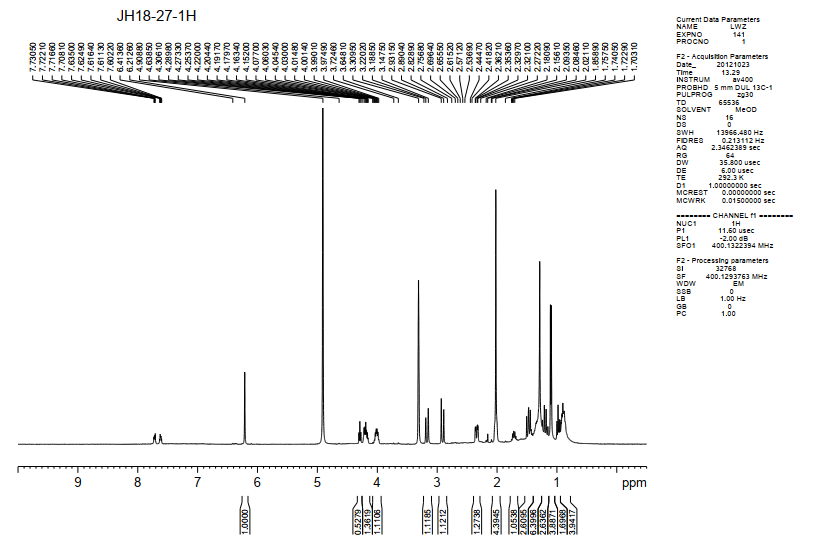


## Figure S5. ^1^H NMR spectrum (400 MHz DMSO-*d*_6_) of spirolone A (1)


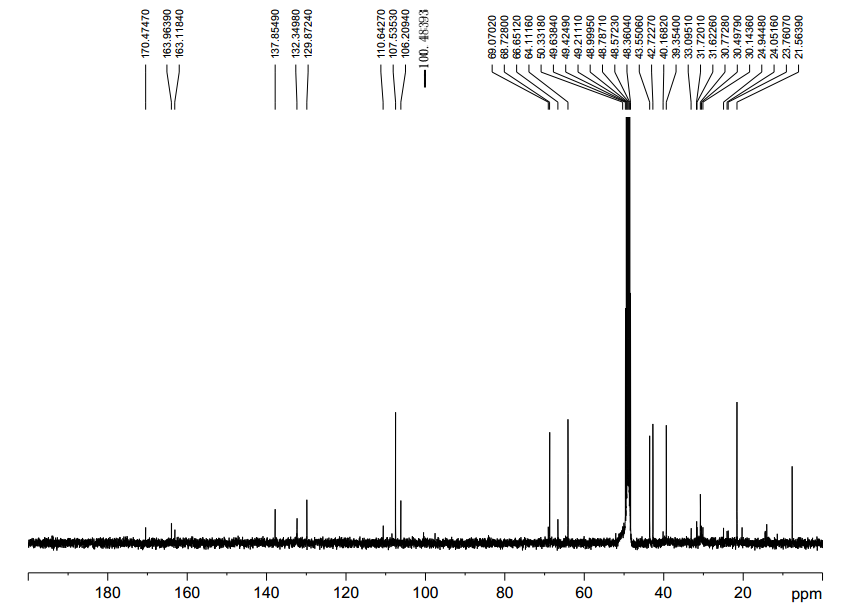


## Figure S6. ^13^C NMR spectrum (100 MHz DMSO-*d*_6_) of spirolone A (1)


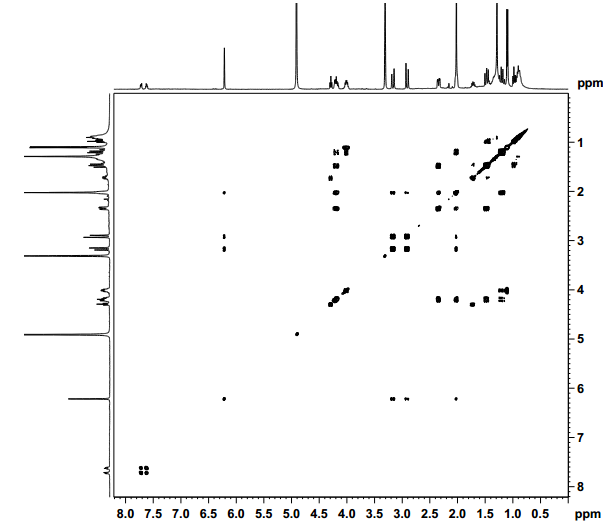


## Figure S7. COSY spectrum (DMSO-*d*_6_) of spirolone A (1)


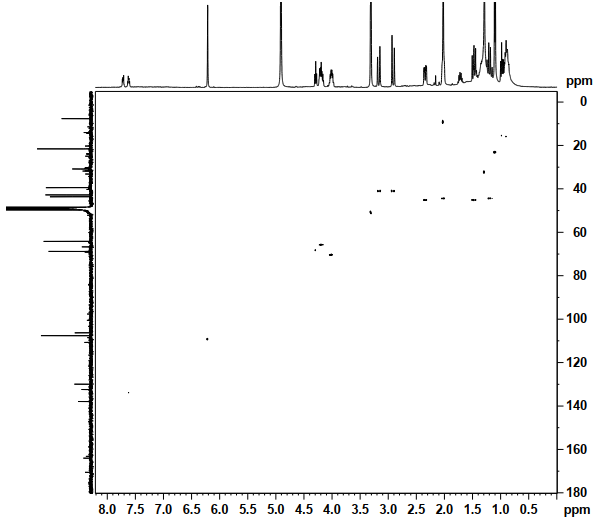


## Figure S8. HSQC spectrum (DMSO-*d*_6_) of spirolone A (1)


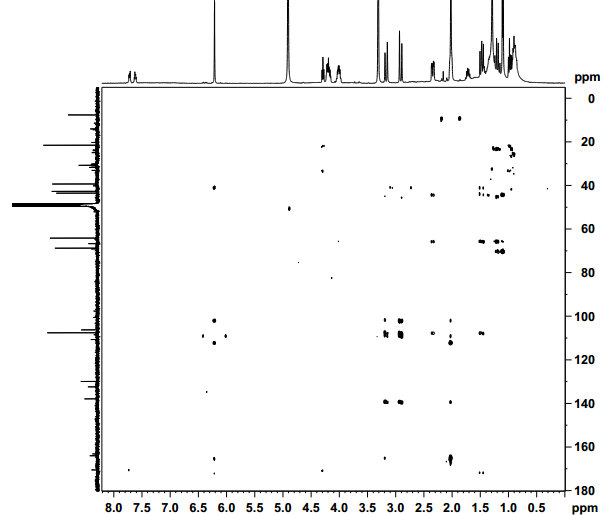


## Figure S9. HMBC spectrum (DMSO-*d*_6_) of spirolone A (1)


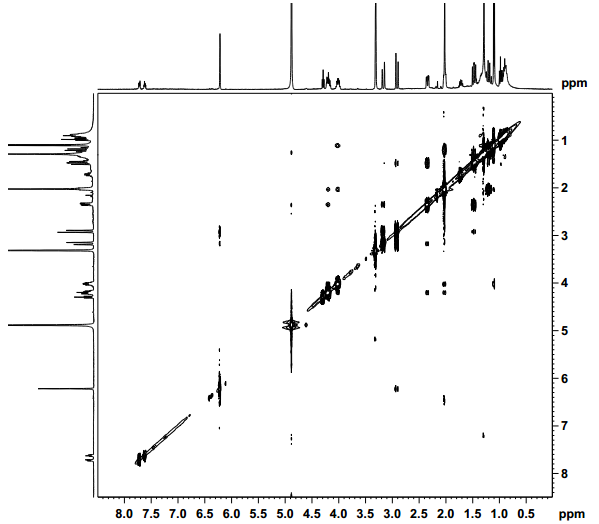


## Figure S10. NOESY spectrum (DMSO-*d*_6_) of spirolone A (1)


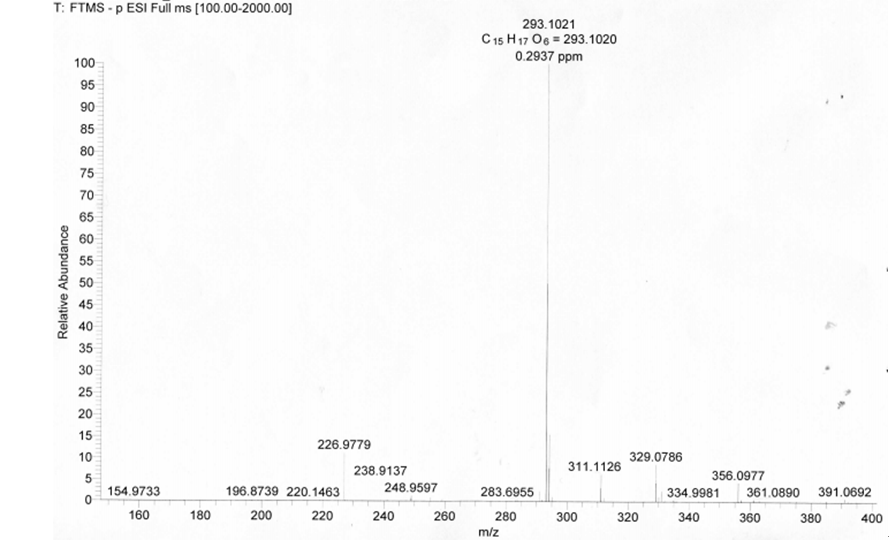


## Figure S11. HRESIMS of spirolone B (2)


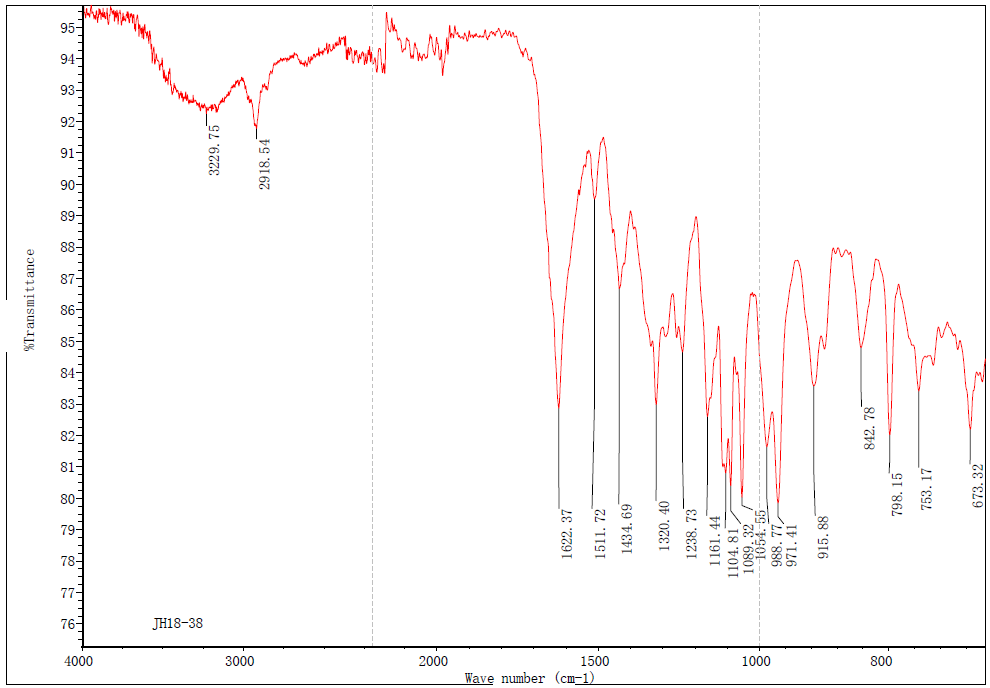


## Figure S12. IR spectrum (ATR approach) of spirolone B (2)


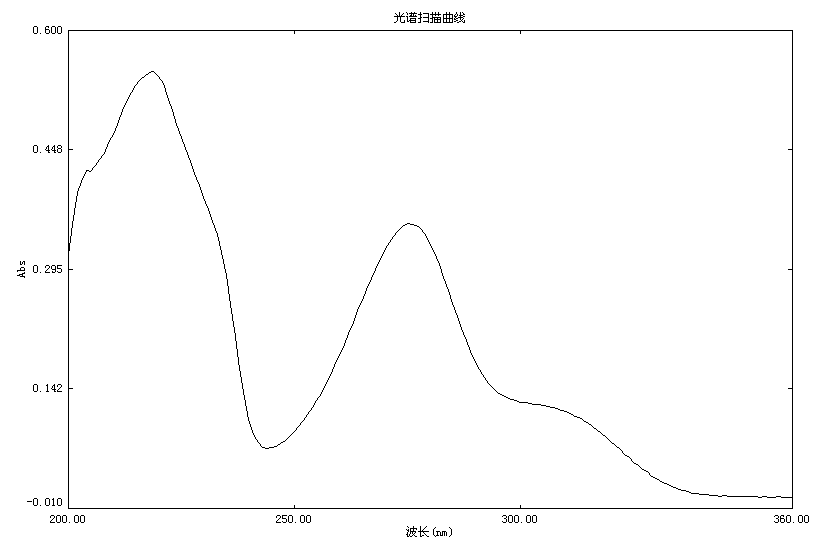


## Figure S13. UV spectrum (MeOH) of spirolone B (2)


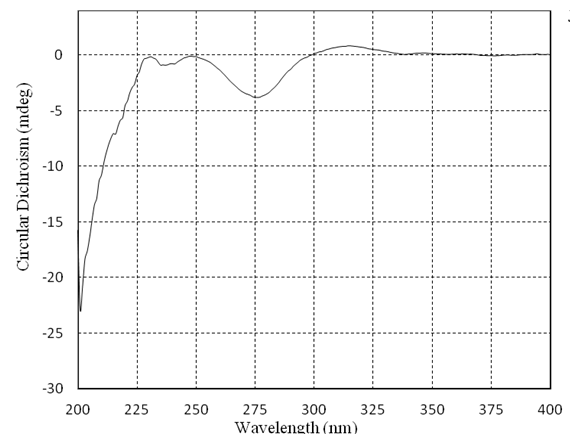


## Figure S14. ECD spectrum (MeOH) of spirolone B (2)


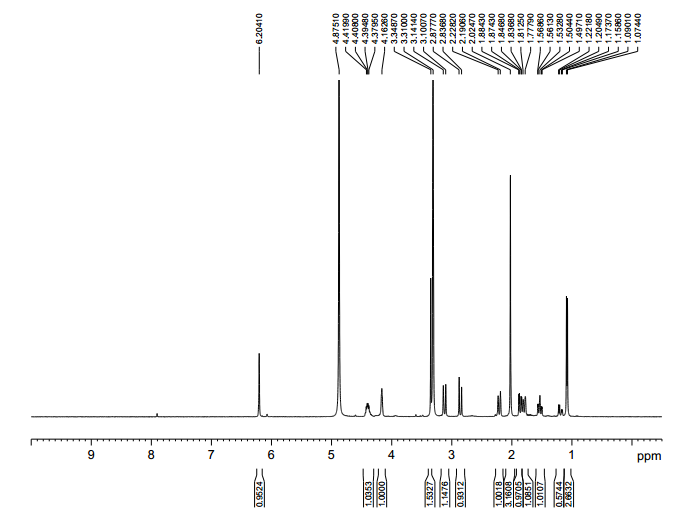


## Figure S15. ^1^H NMR spectrum (400 MHz DMSO-*d*_6_) of spirolone B (2)


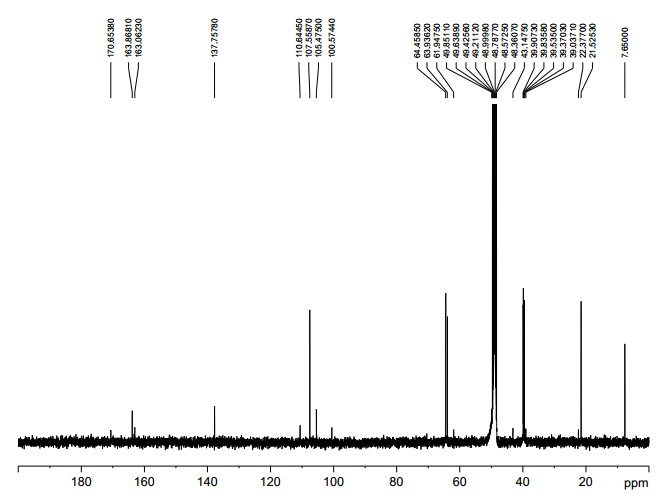


## Figure S16. ^13^C NMR spectrum (100 MHz DMSO-*d*_6_) of spirolone B (2)


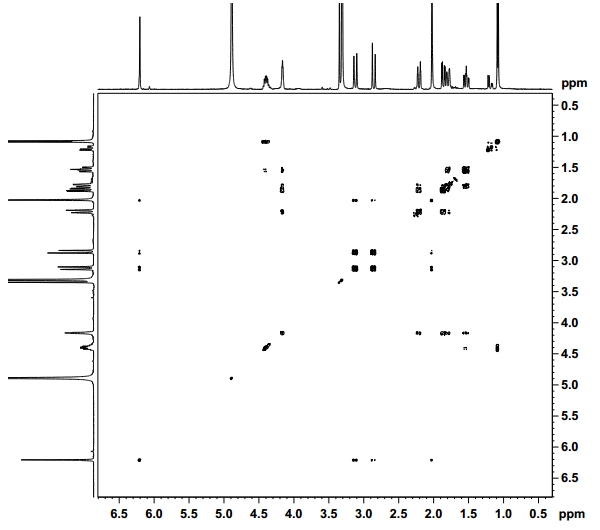


## Figure S17. COSY spectrum (DMSO-*d*_6_) of spirolone B (2)


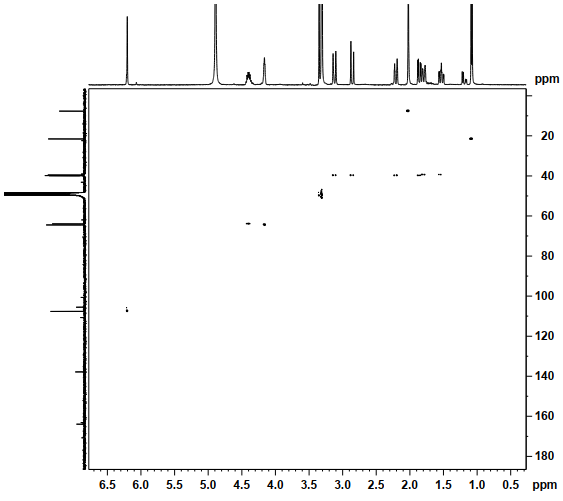


## Figure S18. HSQC spectrum (DMSO-*d*_6_) of spirolone B (2)


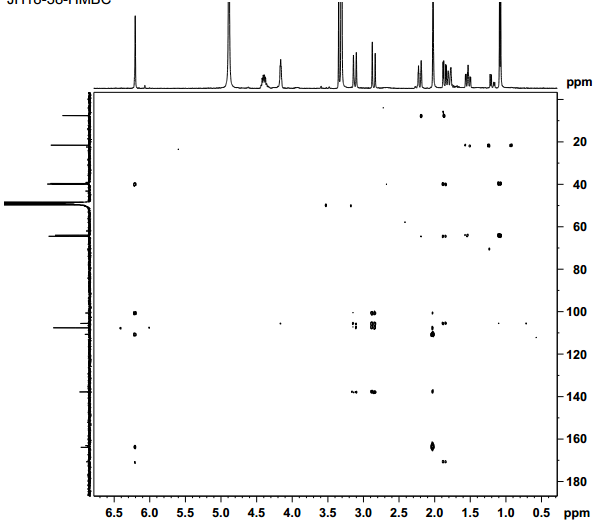


## Figure S19. HMBC spectrum (DMSO-*d*_6_) of spirolone B (2)


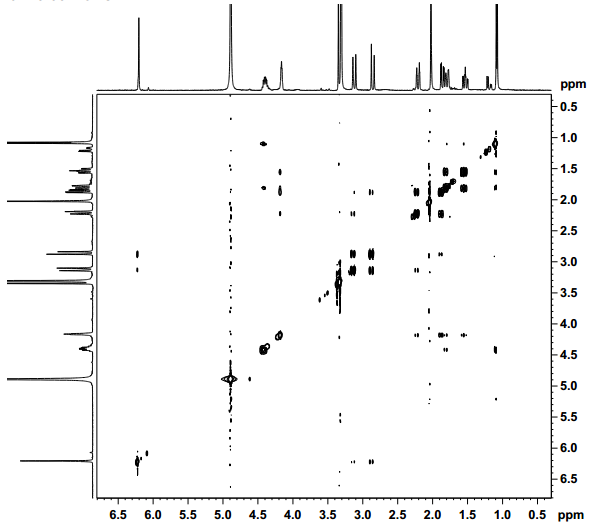


## Figure S20. NOESY spectrum (DMSO-*d*_6_) of spirolone B (2)


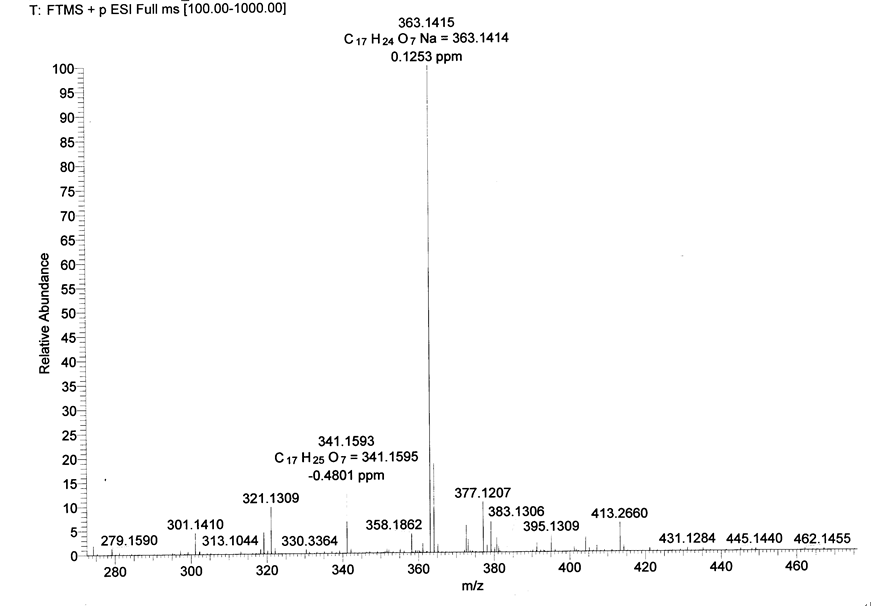


## Figure S21. HRESIMS of spirolone C (3)


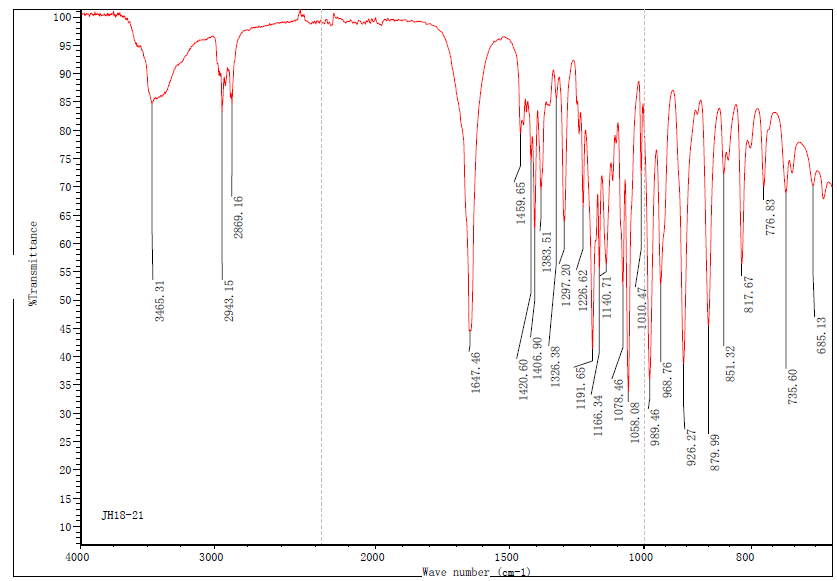


## Figure S22. IR of spirolone C (3)


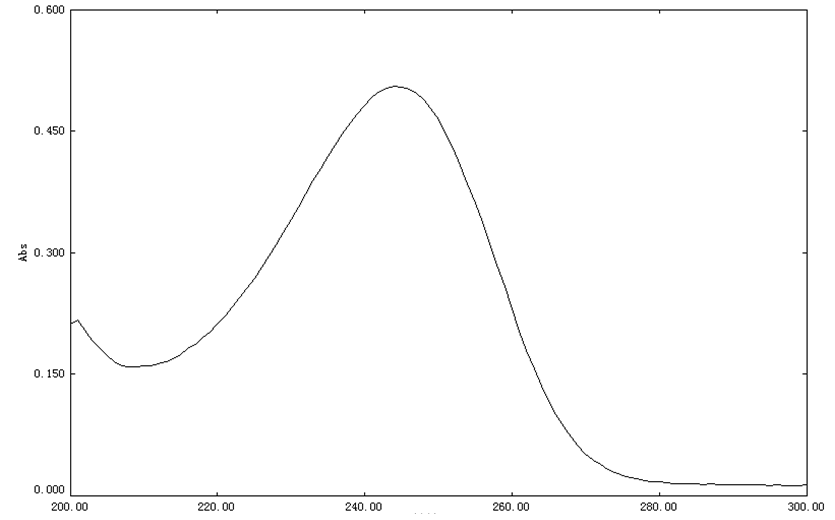


## Figure S23. UV spectrum (MeOH) of spirolone C (3)


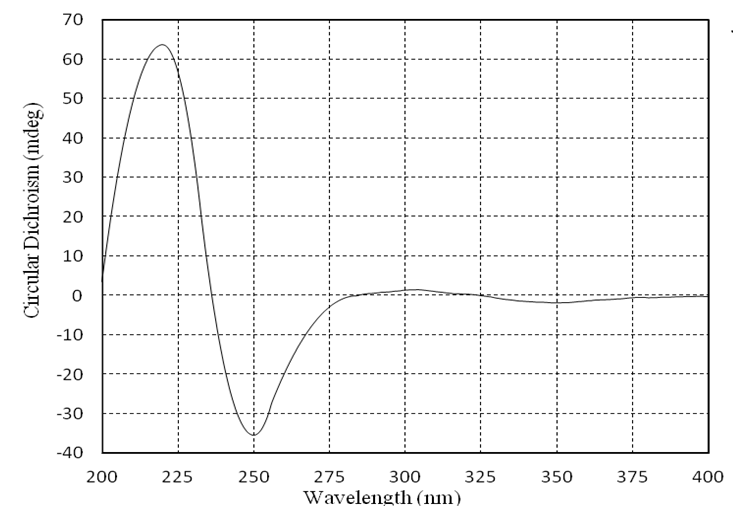


## Figure S24. ECD spectrum (MeOH) of spirolone C (3)


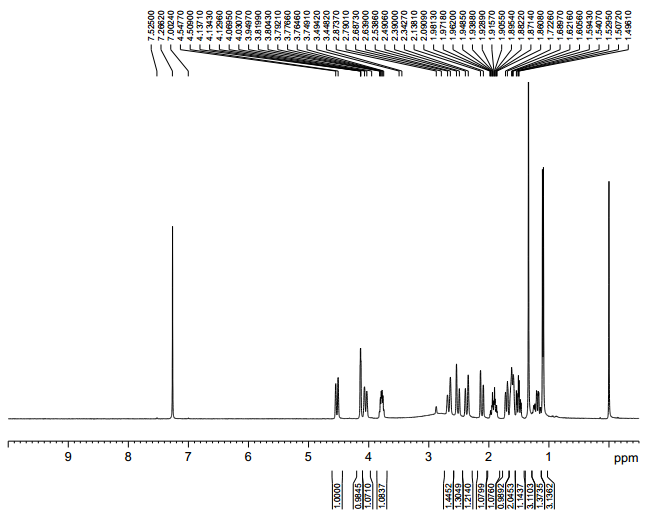


## Figure S25. ^1^H NMR spectrum (400 MHz CDCl_3_) of spirolone C (3)


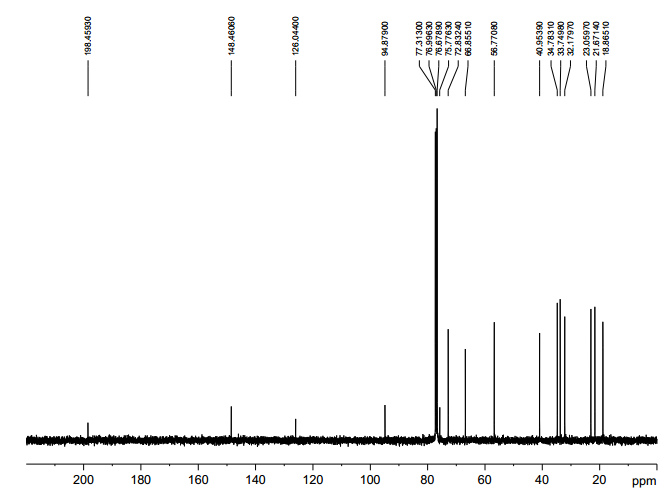


## Figure S26. ^13^C NMR spectrum (100 MHz CDCl_3_) of spirolone C (3)


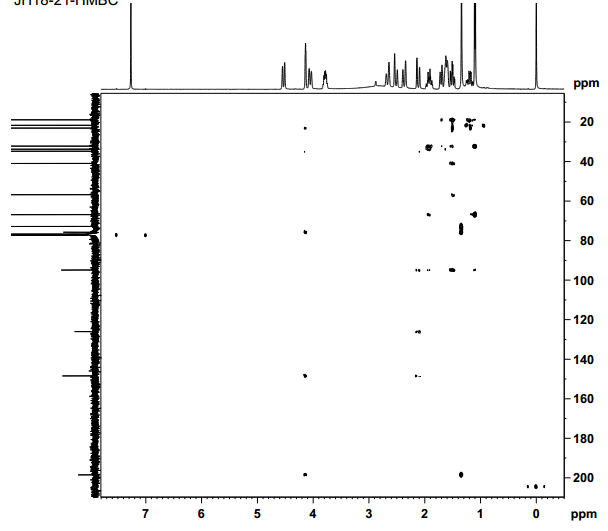


## Figure S27. HMBC spectrum of spirolone C (3)


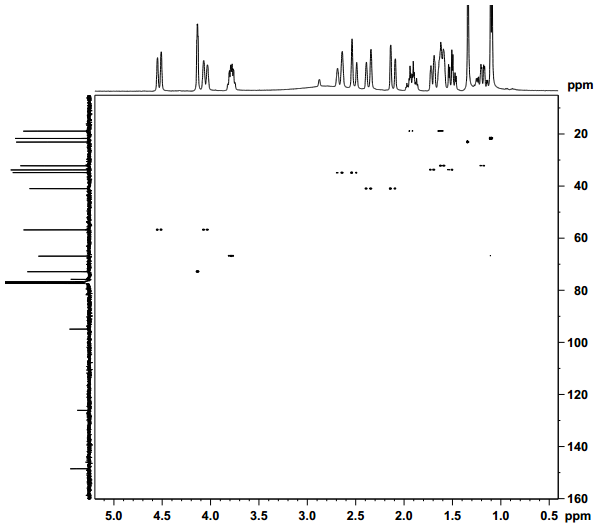


## Figure S28. HSQC of spirolone C (3)


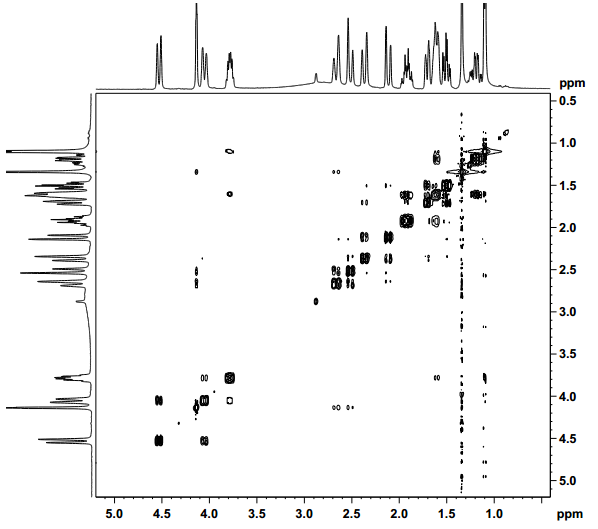


## Figure S29. NOESY spectrum of spirolone C (3)


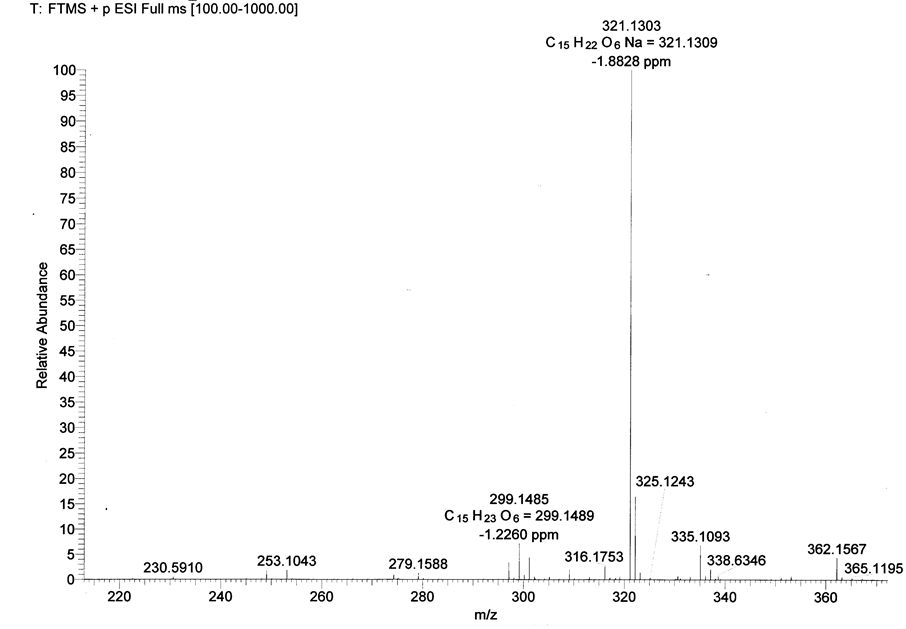


## Figure S30. HRESIMS of spirolone D (4)


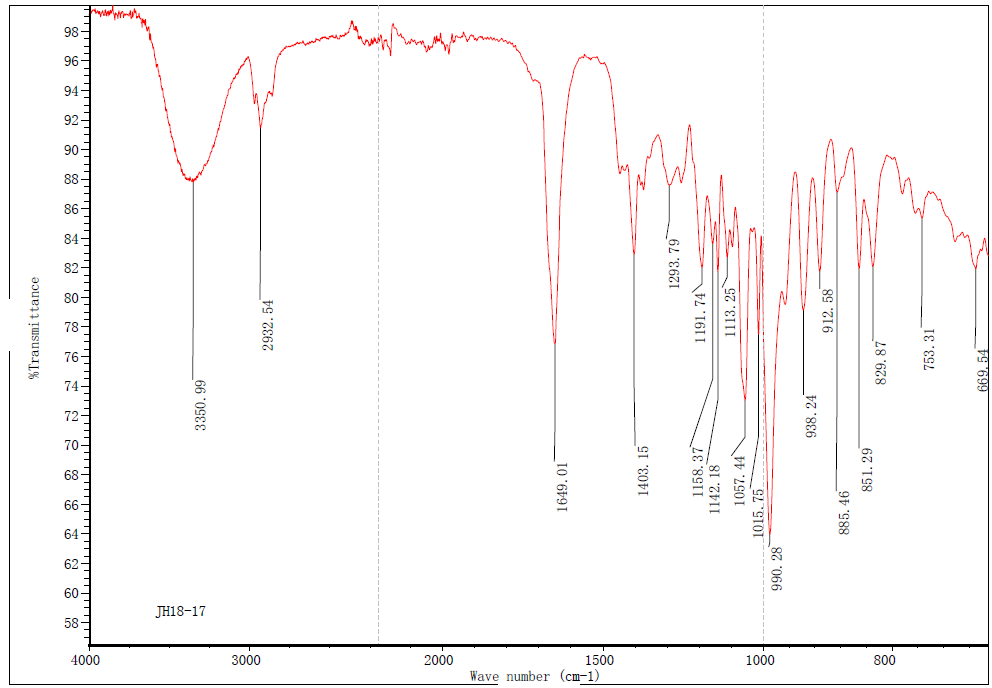


## Figure S31. IR of spirolone D (4)


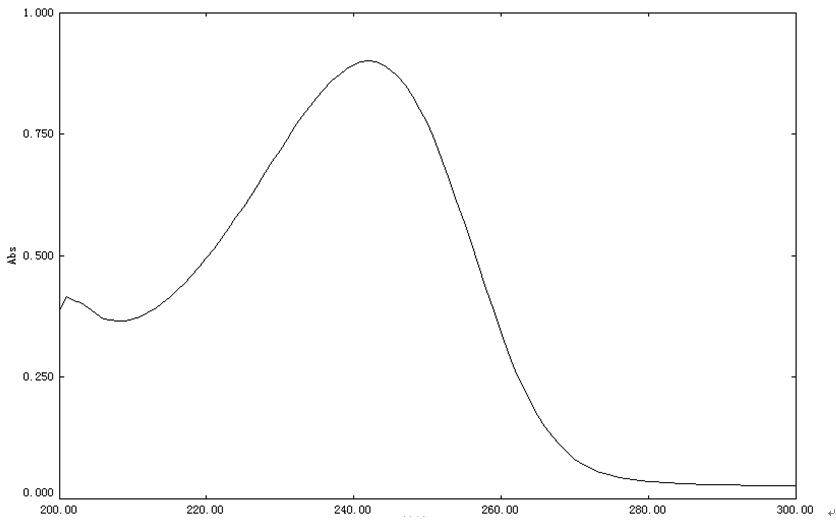


## Figure S32. UV spectrum (MeOH) of spirolone D (4)


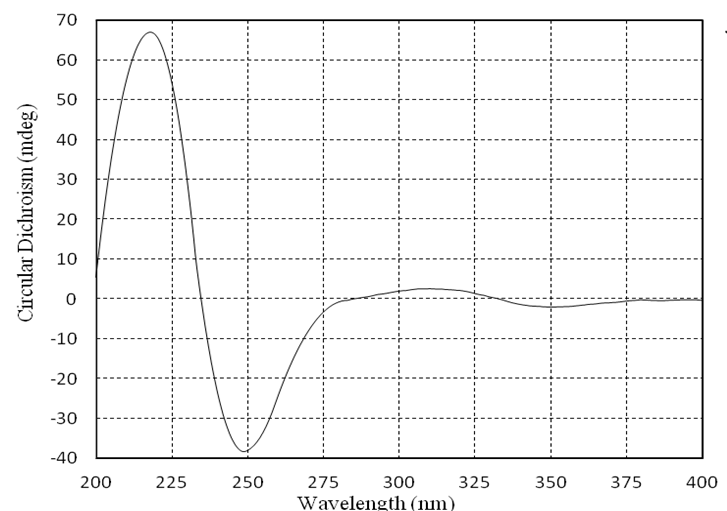


## Figure S33. ECD spectrum (MeOH) of spirolone D (4)

**
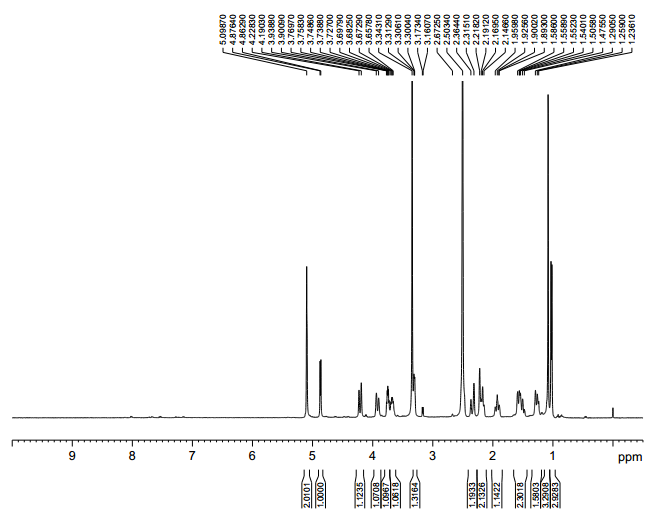
**

## Figure S34. ^1^H NMR spectrum (400 MHz DMSO-*d*_6_) of spirolone D (4)


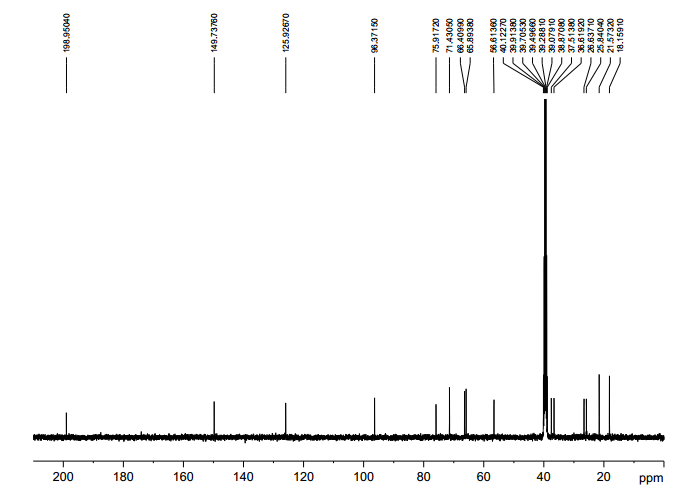


## Figure S35. ^13^C NMR spectrum (100 MHz DMSO-*d*_6_) of spirolone D (4)


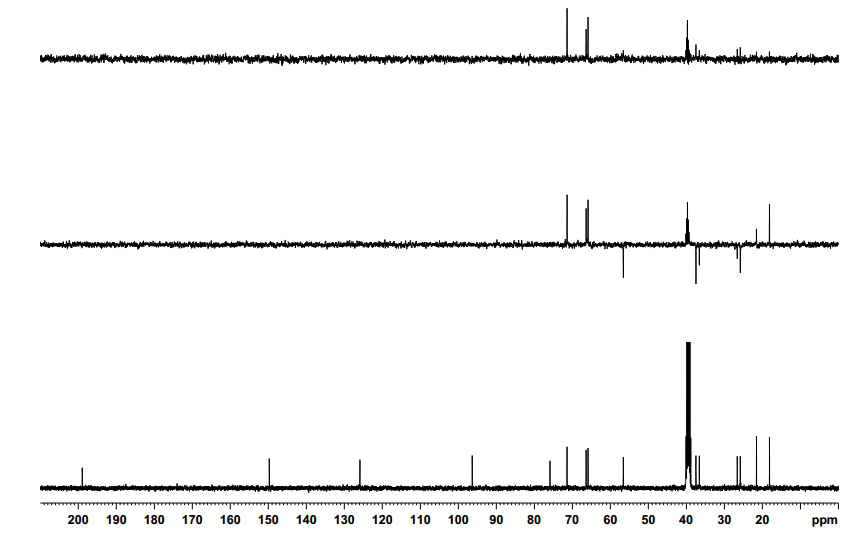


## Figure S36. DEPT spectrum of spirolone D (4)


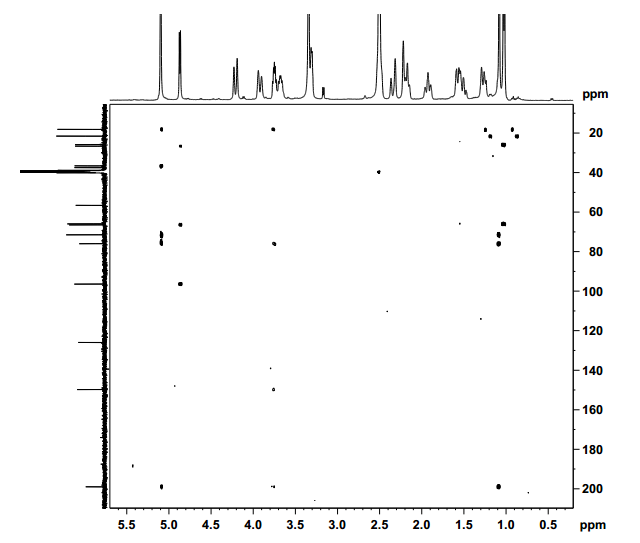


## Figure S37. HMBC of spirolone D (4)


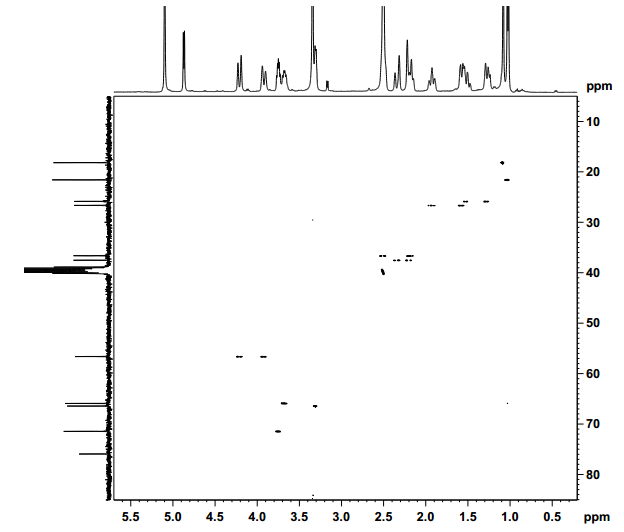


## Figure S38. HSQC of spirolone D (4)


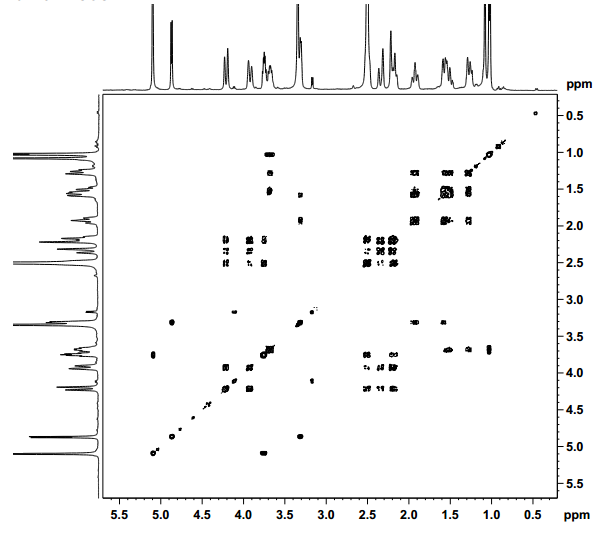


## Figure S39. COSY spectrum of spirolone D (4)


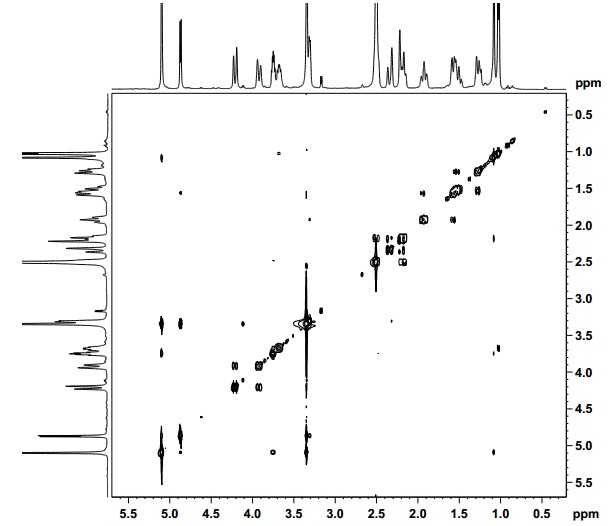


## Figure S40. NOESY spectrum of spirolone D (4)


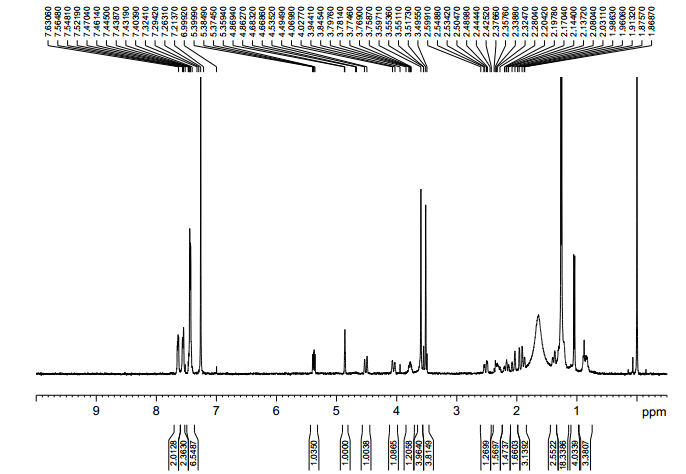


## Figure S41. ^1^H NMR spectrum (400 MHz CDCl_3_) of (*S*)-MTPA ester of 4 (4a)


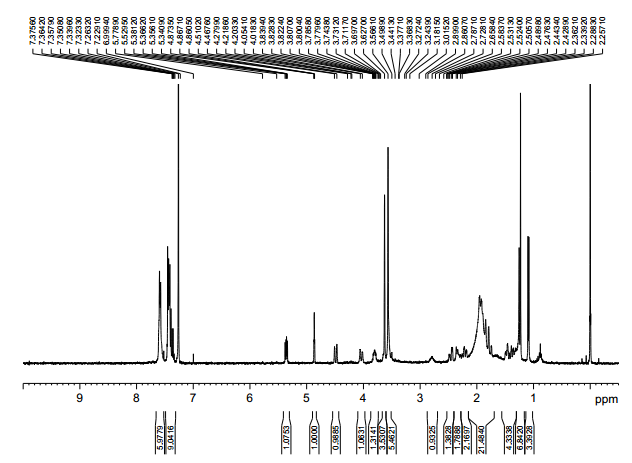


## Figure S42. ^1^H NMR spectrum (400 MHz CDCl_3_) of (*R*)-MTPA ester of 4 (4b)


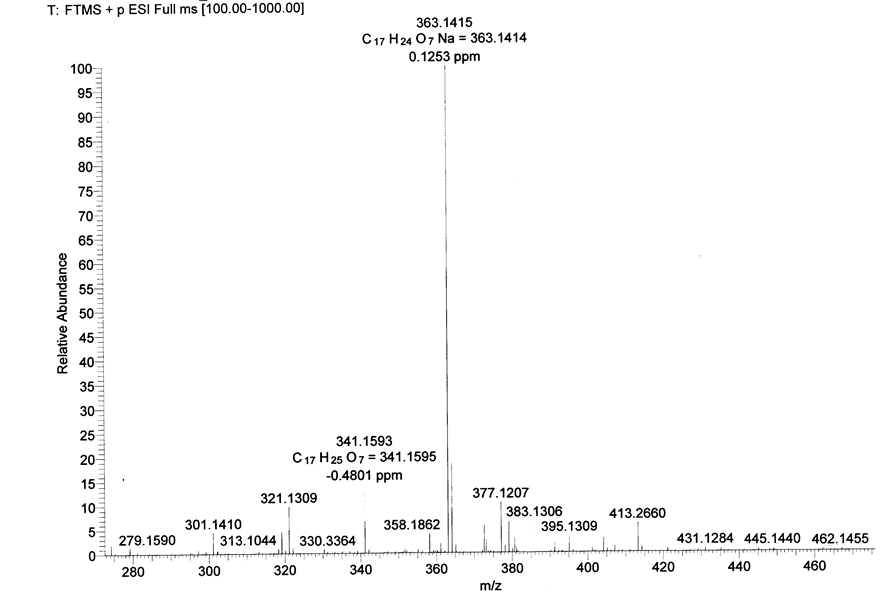


## Figure S43. HRESIMS of spirolone E (5)


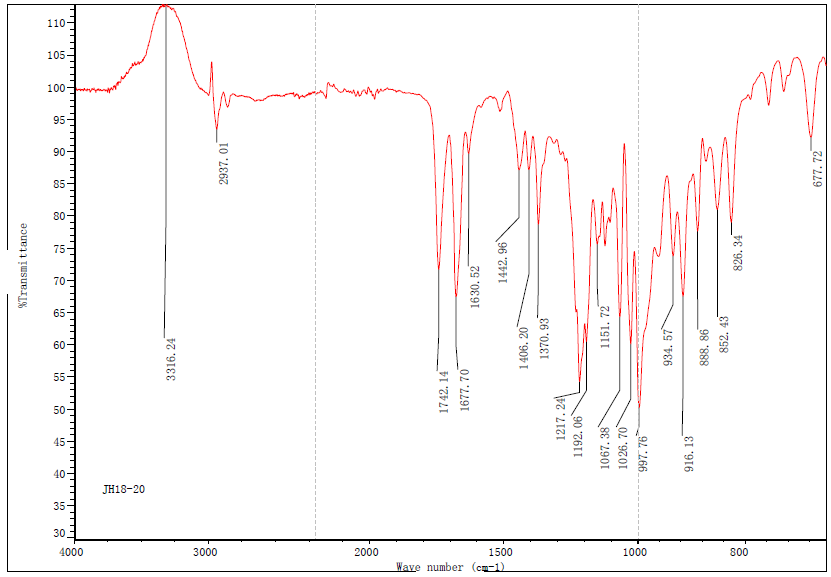


## Figure S44. IR of spirolone E (5)


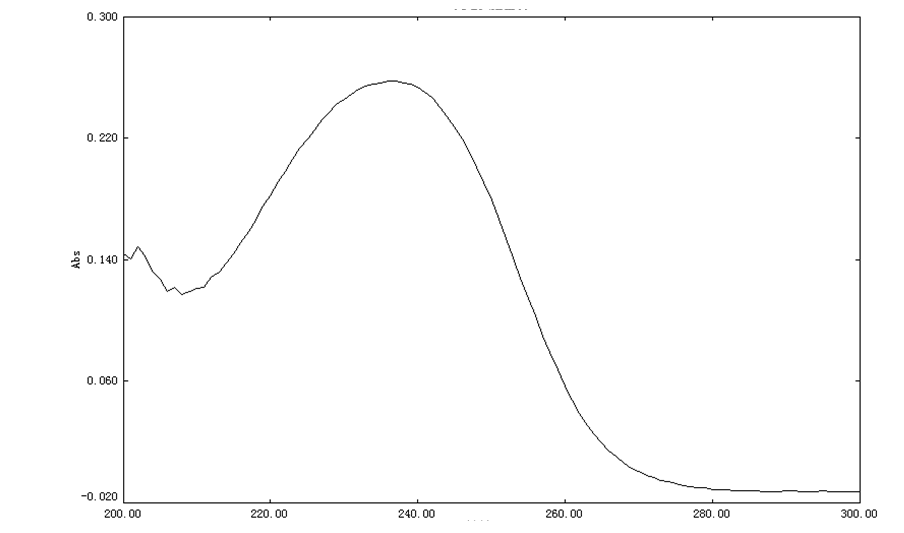


## Figure S45. UV spectrum (MeOH) of spirolone E (5)


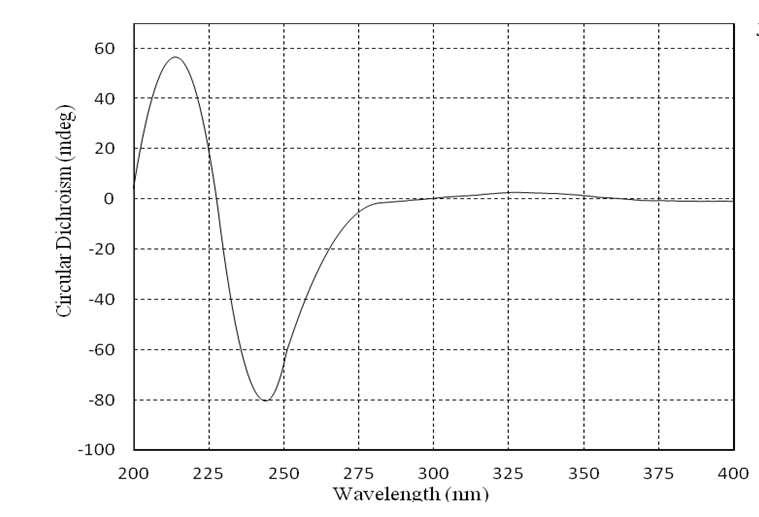


## Figure S46. ECD spectrum (MeOH) of spirolone E (5)


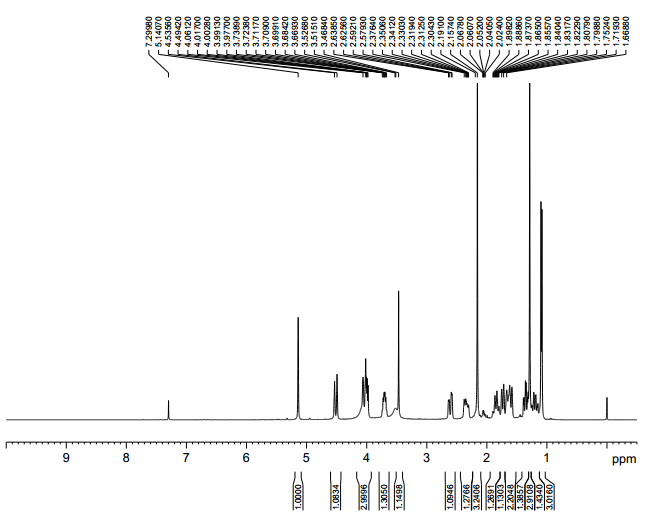


## Figure S47. ^1^H NMR spectrum (400 MHz CDCl_3_) of spirolone E (5)


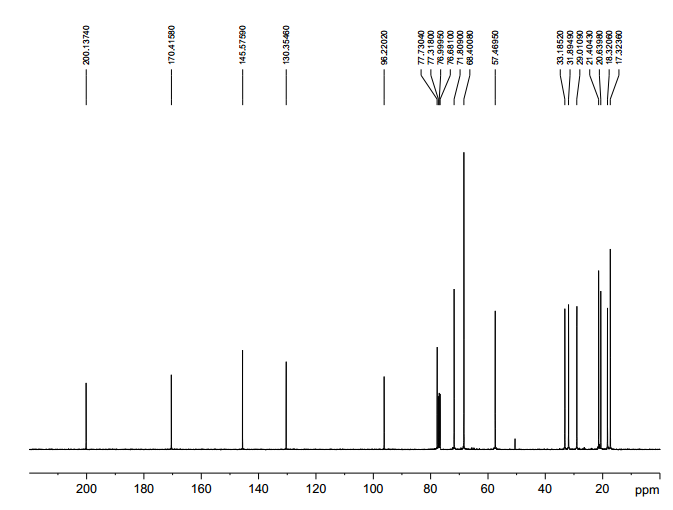


## Figure S48. ^13^C NMR spectrum (100 MHz CDCl_3_) of spirolone E (5)


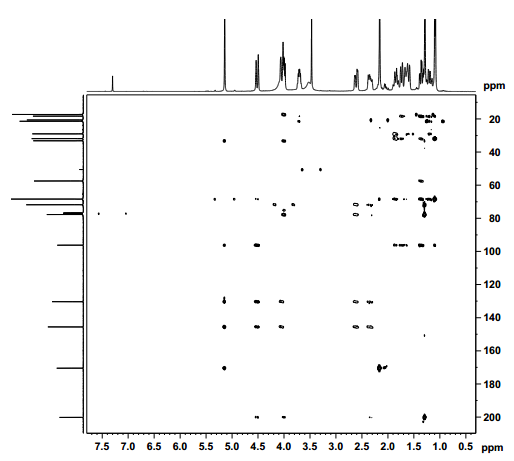


## Figure S49. HMBC of spirolone E (5)


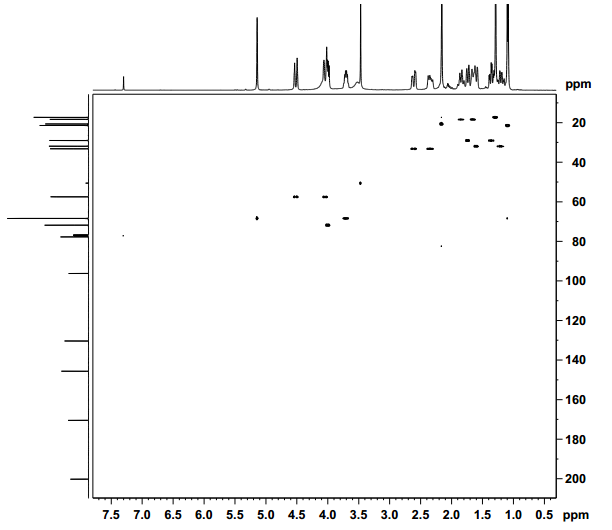


## Figure S50. HSQC of spirolone E (5)


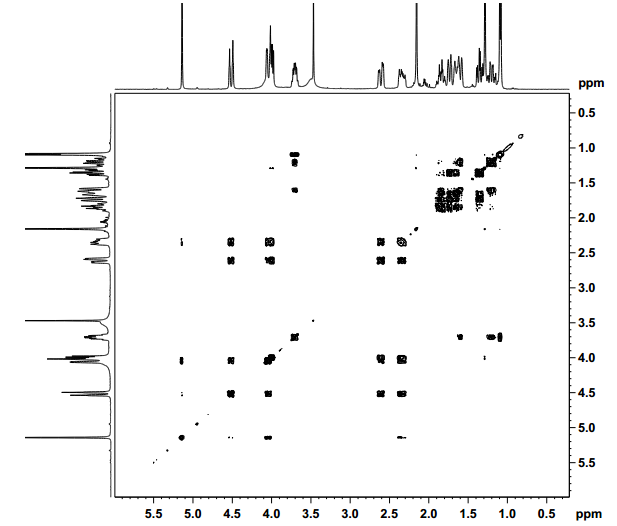


## Figure S51. COSY spectrum of spirolone E (5)


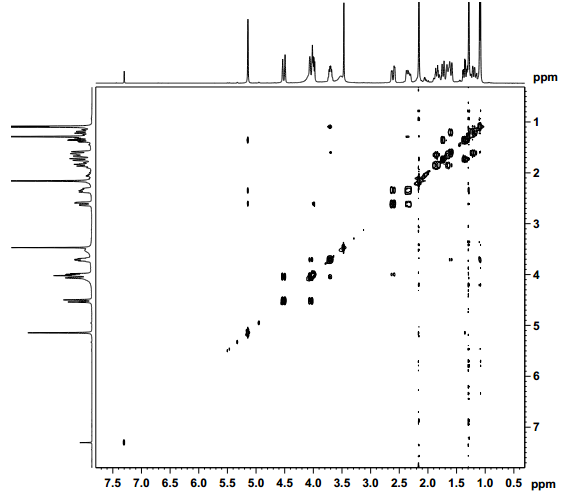


## Figure S52. NOESY spectrum of spirolone E (5)

## Computation Section.

Conformational searches were run by employing the “systematic” procedure implemented in Spartan’14 using MMFF (Merck molecular force field). All MMFF minima were reoptimized with DFT calculations at the B3LYP/6-31+G(d) level using the Gaussian09 program. The geometry was optimized starting from various initial conformations, with vibrational frequency calculations confirming the presence of minima. Time-dependent DFT (TDDFT) calculations were performed on the two lowest-energy conformations for both **4** and three lowest-energy conformations for **5** (>2% population) for each configuration using a polarizable continuum model (PCM) for Methanol. ECD spectra were generated using the program SpecDis^19^.

Cartesian coordinates of the low-energy reoptimized conformers of **5** calculated at B3LYP/6-31+G(d) level of theory.

| Conformer A | | Standard Orientation  (Ångstroms) | | |
| --- | --- | --- | --- | --- |
| I | Atom | X | Y | Z |
| 1 | C | 1.531441 | -0.23672 | 0.440962 |
| 2 | O | 1.140572 | -1.57326 | 0.789011 |
| 3 | C | -0.07036 | -1.99832 | 0.145226 |
| 4 | C | -1.15491 | -0.9598 | 0.186458 |
| 5 | C | -0.95671 | 0.312318 | 0.58533 |
| 6 | C | 0.452117 | 0.756056 | 0.960882 |
| 7 | C | -2.49989 | -1.39132 | -0.29054 |
| 8 | C | -3.56544 | -0.31602 | -0.53003 |
| 9 | C | -3.44878 | 0.748811 | 0.569876 |
| 10 | C | -2.04551 | 1.355147 | 0.642917 |
| 11 | O | -4.87881 | -0.89221 | -0.43178 |
| 12 | C | -3.42449 | 0.234191 | -1.95021 |
| 13 | O | -4.40793 | 1.791049 | 0.366774 |
| 14 | O | -2.73125 | -2.57872 | -0.5225 |
| 15 | C | 2.935828 | 0.005426 | 1.05183 |
| 16 | C | 3.725416 | 1.029722 | 0.251348 |
| 17 | C | 3.87687 | 0.576416 | -1.2003 |
| 18 | C | 2.813414 | -0.45425 | -1.58901 |
| 19 | O | 1.571037 | -0.08414 | -0.99681 |
| 20 | C | 2.624215 | -0.51272 | -3.09802 |
| 21 | O | 0.777728 | 2.104783 | 2.921235 |
| 22 | C | 0.518718 | 0.98465 | 2.471453 |
| 23 | C | 0.263978 | -0.17133 | 3.409573 |
| 24 | H | -0.39278 | -2.90487 | 0.667971 |
| 25 | H | 0.1595 | -2.27599 | -0.88975 |
| 26 | H | 0.631863 | 1.726899 | 0.477559 |
| 27 | H | -3.69163 | 0.294475 | 1.540149 |
| 28 | H | -1.9729 | 1.930857 | 1.573816 |
| 29 | H | -1.89815 | 2.081568 | -0.16603 |
| 30 | H | -4.81043 | -1.78383 | -0.83131 |
| 31 | H | -2.44683 | 0.693393 | -2.12593 |
| 32 | H | -3.547 | -0.56959 | -2.68623 |
| 33 | H | -4.20301 | 0.973215 | -2.16854 |
| 34 | H | -5.25842 | 1.332761 | 0.210514 |
| 35 | H | 2.89033 | 0.297945 | 2.104694 |
| 36 | H | 3.49743 | -0.93961 | 1.057882 |
| 37 | H | 4.713424 | 1.188856 | 0.697294 |
| 38 | H | 3.206789 | 1.995618 | 0.282949 |
| 39 | H | 3.813984 | 1.464381 | -1.84166 |
| 40 | H | 4.872293 | 0.146814 | -1.36481 |
| 41 | H | 3.096693 | -1.45572 | -1.24091 |
| 42 | H | 2.275593 | 0.449301 | -3.48964 |
| 43 | H | 1.856952 | -1.25097 | -3.3556 |
| 44 | H | 3.554905 | -0.78419 | -3.60518 |
| 45 | H | 1.052939 | -0.91951 | 3.316262 |
| 46 | H | 0.263515 | 0.197096 | 4.440089 |
| 47 | H | -0.7123 | -0.61866 | 3.212751 |

| Conformer B | | Standard Orientation  (Ångstroms) | | |
| --- | --- | --- | --- | --- |
| I | Atom | X | Y | Z |
| 1 | C | 1.447191 | 0.271614 | 0.308017 |
| 2 | O | 1.420869 | -1.15102 | 0.485473 |
| 3 | C | 0.340087 | -1.79252 | -0.20781 |
| 4 | C | -0.97356 | -1.08897 | -0.0247 |
| 5 | C | -1.0943 | 0.131038 | 0.534554 |
| 6 | C | 0.165745 | 0.877748 | 0.951676 |
| 7 | C | -2.18009 | -1.79644 | -0.5409 |
| 8 | C | -3.49884 | -1.01957 | -0.62302 |
| 9 | C | -3.6116 | -0.10515 | 0.605215 |
| 10 | C | -2.41223 | 0.836014 | 0.738826 |
| 11 | O | -4.61085 | -1.92966 | -0.58373 |
| 12 | C | -3.57316 | -0.28137 | -1.96058 |
| 13 | O | -4.81679 | 0.664262 | 0.552938 |
| 14 | O | -2.10427 | -2.96346 | -0.92758 |
| 15 | C | 2.730147 | 0.830706 | 0.948703 |
| 16 | C | 4.000885 | 0.296801 | 0.286877 |
| 17 | C | 3.749138 | -0.24923 | -1.11063 |
| 18 | C | 2.632256 | 0.497598 | -1.83326 |
| 19 | O | 1.41992 | 0.625447 | -1.08742 |
| 20 | C | 3.061543 | 1.894319 | -2.27575 |
| 21 | O | 0.320681 | 2.032768 | 3.052702 |
| 22 | C | 0.247125 | 0.942675 | 2.478104 |
| 23 | C | 0.244727 | -0.33945 | 3.276653 |
| 24 | H | 0.284127 | -2.8114 | 0.189349 |
| 25 | H | 0.594982 | -1.8643 | -1.27116 |
| 26 | H | 0.06872 | 1.911673 | 0.59 |
| 27 | H | -3.6823 | -0.72311 | 1.510734 |
| 28 | H | -2.44897 | 1.293208 | 1.735331 |
| 29 | H | -2.4953 | 1.668172 | 0.028635 |
| 30 | H | -4.33311 | -2.71436 | -1.09984 |
| 31 | H | -2.75792 | 0.437529 | -2.08749 |
| 32 | H | -3.51718 | -0.99213 | -2.79393 |
| 33 | H | -4.52647 | 0.247023 | -2.06979 |
| 34 | H | -5.52563 | 0.021088 | 0.348764 |
| 35 | H | 2.728402 | 1.924718 | 0.862196 |
| 36 | H | 2.790613 | 0.591421 | 2.015841 |
| 37 | H | 4.440058 | -0.49627 | 0.904312 |
| 38 | H | 4.747379 | 1.099421 | 0.249391 |
| 39 | H | 4.676383 | -0.23569 | -1.69512 |
| 40 | H | 3.464391 | -1.30528 | -1.02299 |
| 41 | H | 2.376928 | -0.06505 | -2.73901 |
| 42 | H | 3.269406 | 2.544258 | -1.42009 |
| 43 | H | 2.256555 | 2.375897 | -2.8419 |
| 44 | H | 3.953477 | 1.853809 | -2.90842 |
| 45 | H | 1.131131 | -0.93398 | 3.050191 |
| 46 | H | 0.261708 | -0.09679 | 4.343689 |
| 47 | H | -0.66201 | -0.91401 | 3.076961 |

| Conformer C | | Standard Orientation  (Ångstroms) | | |
| --- | --- | --- | --- | --- |
| I | Atom | X | Y | Z |
| 1 | C | 1.455296 | -0.41329 | 0.53444 |
| 2 | O | 1.085656 | -1.76996 | 0.799016 |
| 3 | C | -0.08724 | -2.2015 | 0.100678 |
| 4 | C | -1.19582 | -1.1901 | 0.103879 |
| 5 | C | -1.05058 | 0.079749 | 0.530895 |
| 6 | C | 0.322453 | 0.552421 | 0.994633 |
| 7 | C | -2.50608 | -1.65157 | -0.43903 |
| 8 | C | -3.598 | -0.60583 | -0.68948 |
| 9 | C | -3.55261 | 0.437865 | 0.434603 |
| 10 | C | -2.17329 | 1.085518 | 0.56428 |
| 11 | O | -4.89599 | -1.22286 | -0.65279 |
| 12 | C | -3.42619 | -0.02012 | -2.09251 |
| 13 | O | -4.53408 | 1.457758 | 0.216764 |
| 14 | O | -2.68973 | -2.83569 | -0.72592 |
| 15 | C | 2.806641 | -0.17607 | 1.257731 |
| 16 | C | 3.643687 | 0.878646 | 0.550689 |
| 17 | C | 3.917836 | 0.461694 | -0.89444 |
| 18 | C | 2.887474 | -0.55311 | -1.3991 |
| 19 | O | 1.604353 | -0.19783 | -0.89011 |
| 20 | C | 2.813913 | -0.55889 | -2.91918 |
| 21 | O | -0.08681 | -0.17293 | 3.278225 |
| 22 | C | 0.274241 | 0.722073 | 2.513789 |
| 23 | C | 0.660742 | 2.078488 | 3.05094 |
| 24 | H | -0.41403 | -3.12516 | 0.589808 |
| 25 | H | 0.194285 | -2.45415 | -0.92809 |
| 26 | H | 0.504885 | 1.536778 | 0.542632 |
| 27 | H | -3.81488 | -0.04209 | 1.387226 |
| 28 | H | -2.15356 | 1.650147 | 1.504383 |
| 29 | H | -2.02025 | 1.828844 | -0.22813 |
| 30 | H | -4.77511 | -2.11759 | -1.03331 |
| 31 | H | -2.45697 | 0.470203 | -2.22573 |
| 32 | H | -3.50116 | -0.81049 | -2.84918 |
| 33 | H | -4.21818 | 0.701241 | -2.3213 |
| 34 | H | -5.365 | 0.974964 | 0.030049 |
| 35 | H | 2.676783 | 0.073383 | 2.314569 |
| 36 | H | 3.37941 | -1.11462 | 1.274799 |
| 37 | H | 4.590171 | 1.038268 | 1.078793 |
| 38 | H | 3.110735 | 1.836978 | 0.561687 |
| 39 | H | 3.913914 | 1.36614 | -1.51547 |
| 40 | H | 4.921921 | 0.030133 | -0.98422 |
| 41 | H | 3.143541 | -1.56663 | -1.06513 |
| 42 | H | 2.496213 | 0.416981 | -3.30275 |
| 43 | H | 2.068495 | -1.28577 | -3.25985 |
| 44 | H | 3.780571 | -0.81523 | -3.36298 |
| 45 | H | -0.06479 | 2.82367 | 2.715959 |
| 46 | H | 0.665941 | 2.053195 | 4.144174 |
| 47 | H | 1.661188 | 2.346318 | 2.704056 |

Cartesian coordinates of the low-energy reoptimized conformers of **4** calculated at B3LYP/6-31+G(d) level of theory.

| Conformer A | | Standard Orientation  (Ångstroms) | | |
| --- | --- | --- | --- | --- |
| I | Atom | X | Y | Z |
| 1 | C | 1.499746 | 0.619668 | 0.369126 |
| 2 | O | 1.077614 | 0.710047 | 1.742962 |
| 3 | C | -0.15331 | 0.013196 | 1.989285 |
| 4 | C | -1.21171 | 0.325961 | 0.97294 |
| 5 | C | -0.96324 | 0.968035 | -0.18403 |
| 6 | C | 0.461611 | 1.346001 | -0.52642 |
| 7 | C | -2.59537 | -0.13039 | 1.286733 |
| 8 | C | -3.6575 | -0.03792 | 0.184713 |
| 9 | C | -3.43683 | 1.259153 | -0.6074 |
| 10 | C | -2.02258 | 1.350218 | -1.18343 |
| 11 | O | -4.9702 | 0.033047 | 0.767233 |
| 12 | C | -3.61813 | -1.30621 | -0.66952 |
| 13 | O | -4.3914 | 1.372872 | -1.66824 |
| 14 | O | -2.86619 | -0.60593 | 2.390444 |
| 15 | C | 2.881579 | 1.347598 | 0.271863 |
| 16 | C | 3.701353 | 0.794568 | -0.88472 |
| 17 | C | 3.885138 | -0.72129 | -0.75633 |
| 18 | C | 2.828422 | -1.36982 | 0.145751 |
| 19 | O | 1.564385 | -0.74831 | -0.07328 |
| 20 | C | 2.683188 | -2.85483 | -0.1601 |
| 21 | O | 3.648449 | 1.157249 | 1.476675 |
| 22 | H | -0.48158 | 0.319345 | 2.988055 |
| 23 | H | 0.047166 | -1.06376 | 2.021882 |
| 24 | H | 0.542648 | 2.432767 | -0.40038 |
| 25 | H | 0.656134 | 1.105776 | -1.57867 |
| 26 | H | -3.61007 | 2.119827 | 0.052887 |
| 27 | H | -1.85732 | 2.379192 | -1.52744 |
| 28 | H | -1.92917 | 0.719671 | -2.07641 |
| 29 | H | -4.94379 | -0.54971 | 1.554161 |
| 30 | H | -2.64904 | -1.45331 | -1.15608 |
| 31 | H | -3.81281 | -2.19067 | -0.05094 |
| 32 | H | -4.39763 | -1.29068 | -1.43898 |
| 33 | H | -5.25767 | 1.181226 | -1.25482 |
| 34 | H | 2.747421 | 2.430262 | 0.177405 |
| 35 | H | 3.1976 | 1.020672 | -1.83206 |
| 36 | H | 4.68833 | 1.270054 | -0.92321 |
| 37 | H | 4.882488 | -0.95186 | -0.36244 |
| 38 | H | 3.849344 | -1.15008 | -1.7658 |
| 39 | H | 3.099799 | -1.27301 | 1.203699 |
| 40 | H | 2.344006 | -3.01427 | -1.18964 |
| 41 | H | 1.925341 | -3.30391 | 0.49114 |
| 42 | H | 3.628828 | -3.38532 | -0.01344 |
| 43 | H | 2.984927 | 1.124869 | 2.19682 |

| Conformer B | | Standard Orientation  (Ångstroms) | | |
| --- | --- | --- | --- | --- |
| I | Atom | X | Y | Z |
| 1 | C | 1.443588 | 0.52854 | -0.11207 |
| 2 | O | 1.338558 | 0.495362 | 1.32347 |
| 3 | C | 0.201947 | -0.2527 | 1.78024 |
| 4 | C | -1.06121 | 0.109334 | 1.058075 |
| 5 | C | -1.08557 | 0.855997 | -0.06135 |
| 6 | C | 0.223277 | 1.309074 | -0.67165 |
| 7 | C | -2.33352 | -0.41582 | 1.628167 |
| 8 | C | -3.61759 | -0.25777 | 0.805148 |
| 9 | C | -3.5931 | 1.108902 | 0.10477 |
| 10 | C | -2.34532 | 1.29139 | -0.76145 |
| 11 | O | -4.76532 | -0.27666 | 1.671354 |
| 12 | C | -3.761 | -1.44547 | -0.14824 |
| 13 | O | -4.76344 | 1.284251 | -0.70114 |
| 14 | O | -2.34316 | -0.99828 | 2.713806 |
| 15 | C | 2.753646 | 1.280073 | -0.47182 |
| 16 | C | 3.998828 | 0.524661 | 0.00142 |
| 17 | C | 3.726055 | -0.95125 | 0.262554 |
| 18 | C | 2.652532 | -1.52715 | -0.65783 |
| 19 | O | 1.430486 | -0.7873 | -0.68231 |
| 20 | C | 3.142205 | -1.68287 | -2.09546 |
| 21 | O | 2.788856 | 2.595104 | 0.094897 |
| 22 | H | 0.10485 | -0.0371 | 2.849405 |
| 23 | H | 0.414771 | -1.32266 | 1.676037 |
| 24 | H | 0.317387 | 2.381401 | -0.46255 |
| 25 | H | 0.179744 | 1.174588 | -1.75952 |
| 26 | H | -3.62186 | 1.903912 | 0.862319 |
| 27 | H | -2.27152 | 2.350665 | -1.03877 |
| 28 | H | -2.44857 | 0.743175 | -1.70616 |
| 29 | H | -4.5563 | -0.92451 | 2.375621 |
| 30 | H | -2.9258 | -1.52105 | -0.85126 |
| 31 | H | -3.80263 | -2.38569 | 0.414851 |
| 32 | H | -4.69449 | -1.38622 | -0.71834 |
| 33 | H | -5.51248 | 1.036423 | -0.12206 |
| 34 | H | 2.812022 | 1.396653 | -1.56058 |
| 35 | H | 4.776196 | 0.622383 | -0.76723 |
| 36 | H | 4.425122 | 0.979569 | 0.904186 |
| 37 | H | 3.387384 | -1.05818 | 1.300883 |
| 38 | H | 4.657025 | -1.52516 | 0.187578 |
| 39 | H | 2.395295 | -2.52889 | -0.29372 |
| 40 | H | 3.355193 | -0.71496 | -2.55934 |
| 41 | H | 2.367788 | -2.15611 | -2.70929 |
| 42 | H | 4.045485 | -2.29879 | -2.14242 |
| 43 | H | 2.564647 | 2.483139 | 1.039761 |
